# Insight Into Ecology, Metabolic Potential, and the Taxonomic Composition of Bacterial Communities in the Periodic Water Pond on King George Island (Antarctica)

**DOI:** 10.3389/fmicb.2021.708607

**Published:** 2021-10-08

**Authors:** Tomasz Krucon, Lukasz Dziewit, Lukasz Drewniak

**Affiliations:** Department of Environmental Microbiology and Biotechnology, Institute of Microbiology, Faculty of Biology, University of Warsaw, Warsaw, Poland

**Keywords:** King George Island, psychrotolerants, metabolic properties, Antarctica, bacterial diversity

## Abstract

Polar regions contain a wide variety of lentic ecosystems. These include periodic ponds that have a significant impact on carbon and nitrogen cycling in polar environments. This study was conducted to assess the taxonomic and metabolic diversity of bacteria found in Antarctic pond affected by penguins and sea elephants and to define their role in ongoing processes. Metabolic assays showed that of the 168 tested heterotrophic bacteria present in the Antarctic periodic pond, 96% are able to degrade lipids, 30% cellulose, 26% proteins, and 26% starch. The taxonomic classification of the obtained isolates differs from that based on the composition of the 16S rRNA relative abundances in the studied pond. The dominant *Actinobacteria* constituting 45% of isolates represents a low proportion of the community, around 4%. With the addition of run-off, the proportions of inhabiting bacteria changed, including a significant decrease in the abundance of *Cyanobacteria*, from 2.38 to 0.33%, increase of *Firmicutes* from 9.32 to 19.18%, and a decreasing richness (Chao1 index from 1299 to 889) and diversity (Shannon index from 4.73 to 4.20). Comparative studies of communities found in different Antarctic environments indicate a great role for penguins in shaping bacterial populations.

## Introduction

The northern part of the Antarctic Peninsula and the neighboring islands to the north and along the western side, including King George Island – the largest of the South Shetland Islands – experience the mildest climate within the continent. This Maritime Antarctic region is the largest seasonally ice-free area with especially abundant lakes, ponds, and streams (Toro et al., 2007; Vincent et al., 2009; Mink et al., 2014). Aquatic ecosystems constitute an important element of biodiversity and landscape productivity in polar regions. At the highest latitudes, the preservation of most limnetic ecosystems is dependent on the seasonal freezing and thawing cycle. Increased temperatures contribute to the melting of glaciers, snow, or ground ice and the formation as well as replenishment of water reservoirs. These ecosystems contain dilute meltwaters, hypersaline ponds, ultra-oligotrophic and mesotrophic lakes, nitrate-rich brines, waters affected by animals, and algal wetlands. Ponds, in opposition to lakes, may be permanent or seasonal, functionally different from larger water bodies, and indicate greater biotic and environmental amplitudes (McKnight et al., 1999; De Meester et al., 2005; Howard-Williams and Hawes, 2007; Vincent et al., 2009). They have a considerable impact on the energy balance of landscapes and the carbon cycle (Downing, 2010; Abnizova et al., 2012). Due to their size, there is better contact between water and sediment as well as air and water and groundwater, thus high biogeochemical reactivity is achieved. Diversity in size as well as in the number of freeze-thaw cycles, nutrient availability, local food web, or mineral leaching contributes to the high seasonal variability of these habitats and affects the distribution and composition of the microbial community (Izaguirre et al., 1998; Vinocur and Unrein, 2000; Borghini et al., 2011; Archer et al., 2015). Therefore, water reservoirs located even in close vicinity may show differing susceptibility to abiotic factors such as salinity, nutrient content and temperature (De Meester et al., 2005; Lyons et al., 2012). The characteristics of the microbiological community in the aquatic environment are essential to understanding its functioning. An increase in temperature, and a corresponding increase in ice melt and liquid water supply, can contribute to increased available energy for biota, productivity and population growth (Convey and Peck, 2019), and the subsequent increased pressure exerted by the local fauna on aquatic habitats.

Also, the human presence in the Antarctic poses pressures on the environment. Transport, storage, and use of petroleum-based fuels, mainly diesel, carry the risk of hydrocarbon contamination (Wong et al., 2021). According to The Protocol on Environmental Protection to the Antarctic Treaty, non-indigenous species cannot be introduced to the environment (Committee for Environmental Protection [CEP], 2012) and therefore, bioremediation processes must be based on indigenous species (Zakaria et al., 2021). In this context, the natural attenuation potential of native microbial communities may be crucial in removing contaminants and restoring the disturbed environment.

To date, studies of Antarctic ponds have given insight into the structure of communities forming dense microbiological mats (Fernández-Valiente et al., 2001; Peeters et al., 2011, 2012; Sutherland et al., 2020; Jackson et al., 2021), lining mats of benthic zone and sediments (Archer et al., 2015; Callejas et al., 2018), and phytoplankton (Vinocur and Unrein, 2000; Allende and Mataloni, 2013; Archer et al., 2014). A limited study was conducted to characterize microbial communities in water columns (Archer et al., 2016) and determine their metabolic properties (Ferrés et al., 2015). These studies were mainly focused on identifying the taxonomy of cultivated strains as well as the taxonomy of whole communities based on several fingerprinting methods (e.g., DGGE – Denaturing Gradient Gel Electrophoresis, PLFA – Phospholipid Fatty Acid Analysis, or T-RFLP – Terminal restriction fragment length polymorphism). To our knowledge, there is a lack of comprehensive studies on Antarctic ponds that focus on the metabolic properties of cultivated strains and their taxonomy in relation to total biodiversity and biodiversity of other environments. Considering the importance of bacterial communities in shaping glacial ecosystems and their key role in melt ponds, understanding the ecology of these environments requires examining them in the context of their bacterial communities. Past research suggests that bacterial communities in melt ponds may be more sensitive to environmental change than in other aquatic ecosystems (Hu et al., 2020). Therefore, this study provides important insight into the functioning of bacterial communities in a changing eutrophied environment and provides a framework for more detailed studies of metabolism and metabolite fluxes in these water bodies.

Evaluation of the metabolic properties of cultivated bacteria and the diversity of bacterial communities in natural environments is the first step to understanding their functions. In this work, we sought to investigate the ecology of a periodic pond, located on King George Island, greatly influenced by penguins and sea elephants, where the water blooms were found. Therefore, the aim of this study was (i) isolating the bacteria, identifying the taxonomy, and examining their biochemical properties concerning the metabolism of selected C- and N-compounds, which will allow us to verify their potential to metabolize compounds potentially occurring in the pond and identify the groups of microorganisms involved in these processes; (ii) determining the structure of the community of total bacteria in order to examine what part of the population the obtained isolates represent and comparing changes in the structure of microorganisms caused by the supply of nutrients by animals; (iii) assessing the potential origin of bacteria as well as their similarity to communities in other Antarctic environments.

## Materials and Methods

### Sampling and Isolation of Bacterial Strains

In this study, two water samples from a periodic pond located on the Patelnia Peninsula (62°14′4.76″’ S, 58°28′22.29″’W) at the south coast of King George’s Island were collected. Sampling was carried out on 14th of January (T1) and 2nd of February (T2) in 2012. During this period, the average monthly air temperature at the Polish Arctowski Station located 8.3 km from the pond was 2.4°C in January and 2.0°C in February (Araźny et al., 2013). Conductivity at time T1 was 0.415 mS cm^–1^ and 1.848 mS cm^–1^ at time T2 and pH 7.9 and 8.4, respectively. Samples assigned for microbiological analysis were preserved with sterile glycerol at a final concentration of 10%. Samples were stored at −20°C. To isolate bacteria, water samples were diluted in 0.85% solution of NaCl, and then each dilution was spread onto Petri dishes containing Reasoner’s 2A (R2A) solid medium. Plates with 30–300 colonies were used to calculate the total number of bacteria. Pure bacterial cultures were obtained by triple passages of clones from each time.

### Genomic DNA Extraction, Sequencing, and Analysis of 16S rRNA Gene Sequences

To determine the taxonomic identity of the obtained isolates, extracting, sequencing, and analysis of 16S rRNA gene sequences was performed. Total DNA was isolated using the EURx DNA genomic isolation kit. Amplification reactions of the 16S rRNA gene were carried out using 27F (5′-AGAGTTTGATCCTGGCTCAG-3′) and 1492R (5′-GGTTACCTTGTTACGACTT-3′) primers (Lane, 1991) and Taq polymerase (Thermo Fisher Scientific), and sequencing was performed using the following primers: 341F (5′-CCTACGGGAGGCAGCAG-3′), 518R (5′-ATTACCGCGGCTGCTGG-3′) (Muyzer et al., 1993) and 928F (5′-TAAAACTYAAAKGAATTGACGGG-3′) (Weidner et al., 1996). Sequencing reactions were performed in ABI-PRISM 377 apparatus of Applied Biosystems in the Laboratory of DNA Sequencing and Oligonucleotide Synthesis of the Institute of Biochemistry and Biophysics of the Polish Academy of Sciences. For analysis and visualization of obtained nucleotide sequences, FinchTV (Geospisa) software^1^ was used. The taxonomy of the tested strains was determined by the use of the Classifier tool available on the rdp.cme.msu.edu website. Identification of the closest relatives of each isolate was carried out using the blastn suite on the BLAST website (Camacho et al., 2009) and the reference RNA sequences database (O’Leary et al., 2016). A detailed description can be found in Supplementary File 1.

### Metabolic Characteristics of the Isolates

To determine the ability of microorganisms to conduct extracellular amylolysis, cellulolysis, lipolysis, and proteolysis, oxidation of a phenolic substrate by laccases and siderophores production the replica plating method was used. Cells from 1-week-old bacterial cultures grown on a R2A plate were transferred onto appropriate solid agar media (Table 1) using a sterile inoculation loop. Further, the cultures were incubated at 20°C for 7 days. Changes in color or the appearance of transparency around the growth of bacterial colonies indicated a positive test result. Briefly, in the starch hydrolysis assay, iodine solution was added to the plate after 7 days of incubation. If a transparent clear zone appeared around the growth line of the strain, the result was considered as positive. In the cellulolytic activity assay, the appearance of yellowish, clear color around the growth line of the strain was considered a positive result. In the ligninolytic activity assay, the appearance of brownish color around the growth line of the strain was considered a positive result. In the lipolytic and proteolytic activity assay, the appearance of transparency around the growth line of the strain was considered a positive result. In the siderophore production test, the appearance of yellowish halo around the growth line of the strain was considered a positive result. Using the modified Arnow (1937) and Snow (1954) test, the type of produced siderophores, catecholate, or hydroxamate, respectively, was determined. In both tests, 1-week-old culture of each strain was centrifuged on R2A medium, and the supernatant was transferred to a 96-well titration plate. Then, in the case of the first test, 20 μL of 5 N HCl and 100 μL of nitrite – molybdate reagent (NaNO_2_ 10 g, NaMoO_4_ 10 g in 100 mL double-distilled water), and 10 μL of 10 N NaOH were added to each well, resulting in a pinkish-red color in the presence of catechol siderophores. After 5 min of incubation, the absorbance was measured at 515 nm. In the case of the second test, 20 μL of TTC solution and 10 μL of 10 N NaOH were added to each well, resulting in a deep red color. Then, absorbance was measured at 480 nm.

**TABLE 1:** List of media used in this study.

Tested enzymes/metabolites	Medium/buffer (compounds per liter)	References
–	PBS: NaCl 8 g, KCl 0.2 g, Na_2_HPO_4_ 1.44 g, KH_2_PO_4_ 0.245 g	Phosphate-Buffered Saline [PBS], 2006
–	R2A: yeast extract 0.5 g, proteose peptone 0.5 g, casamino acids 0.5 g, glucose 0.5 g, soluble starch 0.5 g, Na-pyruvate 0.3 g, K_2_HPO_4_ 0.3 g, MgSO_4_ x 7H_2_O 0.05 g, agar 15 g, pH 7.2	Ferrés et al., 2015
Amylases	R2A with 10 g soluble starch	–
Celulases	CMC: KH_2_PO_4_ 0.5 g, MgSO_4_ 0.25 g, carboxymethyl cellulose 2.0 g, gelatin 2.0 g; Red Congo dye 0.2 g, pH 6.8–7.2	Teather and Wood, 1982; Gupta et al., 2012
Laccases	R2A with 0.01% guaiacol	Kiiskinen et al., 2004
Lipases	TBT: peptone 5.0 g, yeast extract 3.0 g, agar 12.0 g, tributyrin 10.0 mL, or olive oil 10.0 mL	Willis, 1960
Proteases	R2A with 10.0 mL skimmed milk	–
Siderophores	CAS agar: Chrome Azurol S 60.5 mg, 1.0 mM FeCl_3_ × 6H2O 10.0 mL, HDTMA 72.9 mg, 10× MM9 salts 100.0 mL (KH_2_PO_4_ 3.0 g, NH_4_Cl 10.0 g, H_2_O to 1.0 L), 10× LB medium 30.0 mL, 20% glucose 10.0 mL, 1M MgSO_4_ 2.0 mL, 1M Na_2_SO_4_ 2.0 mL, 0.1 M CaCl_2_ 1.0 M	Schwyn and Neilands, 1987, modified
Alkanes	Bushnell-Haas medium: K_2_HPO_4_ 1.0 g, KH_2_PO_4_ 1.0 g, NH_4_NO_3_ 1.0 g, MgSO_4_ 0.2 g, CaCl_2_ 0.02 g, FeCl_3_ 0.05 g, diesel/hexadecane 10 mL	Busenell and Haas, 1941

*Medium R2A was obtained from BD Bioscience, LB from Biomaxima, and the other chemicals from Sigma-Aldrich company. All chemicals were of reagent grade and used without further purification.*

The ability to degrade diesel-oil and hexadecane was tested on 96-well titration plates. Three days’ cultures of bacteria grown at R2A medium were centrifuged and washed three times with phosphate buffered saline (PBS) buffer (Table 1). Then, dilutions of 10 times PBS were prepared. On the titration plate, 180 μL of Bushnell-Haas medium (Table 1), 20 μL of bacterial suspension in PBS, and 5 μL of filtered n-hexadecane were applied. The medium with PBS was used as a control. Each test was performed in triplicate. Titration plates were incubated at 20°C with shaking at 180 rpm for 7 days. On each day, OD was measured at a wavelength of 600 nm. The ability of microorganisms to produce biosurfactants was determined by the oil spreading test (Morikawa et al., 2000). The Petri dish was filled with water (40 mL), and 40 μl of diesel oil was added. Then, 10 μl of liquid, stationary culture (7 days) on R2A, R2A with vegetable (canola) oil [1% (v/v)], R2A with diesel oil [1% (v/v)], or R2A with n-hexadecane [1% (v/v)] was applied. The formation of an oil-free zone indicated the presence of biosurfactants in the culture.

### Metagenomic DNA Isolation From Water Samples and Sequencing

After the shipment to the laboratory in Poland, the water samples were unfrozen, concentrated five times by centrifugation 10 mL of each sample at 6000 rpm for 5 min. Supernatants were discarded and then total DNA was isolated from pellets using the MoBio Soil Isolation Kit. Isolation was performed in accordance with the kit manufacturer’s instructions. A variable region, V3-V4, of the 16S rRNA gene was amplified using V3-4F (5′-TCGTCGGCAGCGTCAGATGTGTATAAGAGACAGCCTACG GGNGGCWGCAG-3′) and V3-4R (5′-GTCTCGTGGGCTCGG AGATGTGTATAAGAGACAGGCACHVGGGTATCTAATCC-3′) primers (Klindworth et al., 2013) and KAPA HiFi DNA Polymerase (Roche Company). A detailed description can be found in Supplementary File 1. Filtered double-distilled water was used as a negative control for DNA isolation. After DNA isolation and PCR reaction, DNA electrophoresis in 1.5% agarose gel and DNA visualization with ethidium bromide solution were performed. The concentration of DNA after each step was determined with the Qubit fluorometer (Thermo Fisher Scientific). Isolation and amplification of DNA were performed in triplicate, and then the samples were pooled and sequencing. Amplicons sequencing was performed in the Laboratory of DNA Sequencing and Oligonucleotide Synthesis of the Institute of Biochemistry and Biophysics of the Polish Academy of Sciences, using Illumina Miseq technology.

### Analysis of the Taxonomic Structure of Microbiological Communities

Reads from DNA sequencing were qualitatively filtered using the Trimmomatic tool (v.0.39) (Bolger et al., 2014) with SLIDINGWINDOW:4:20, HEADCROP:10, MINLEN:50, and ILLUMINACLIP followed by pair-end assembled using PANDA-seq software (Masella et al., 2012). Next, sequences containing homopolymers longer than eight nucleotides and ambiguous bases were discarded, and the chimeric sequences were identified (chimera.vsearch) and removed using mothur (v.1.39.5) (Schloss et al., 2009). The obtained sequences were assigned to the taxa using kraken2 (2.0.8-beta) (Wood et al., 2019) and the RDP Taxonomy database (v.18) (Cole et al., 2014) and then, using a self-written script, the data from the obtained report was converted to files associated with mothur (v.1.39.5) and phyloseq (v.1.30.0) (McMurdie and Holmes, 2012) package from the R software. A prediction of the metabolic properties based on 16S rRNA gene sequences was performed using Tax4Fun2 software (Wemheuer et al., 2020). Further taxonomic analysis of *Cyanobacteria* was performed after extraction of reads classified into this phylum and their reclassification using kraken2 and a database based on 16S RefSeq NCBI records^2^.

### Statistical Analysis

Statistical analysis was performed in R (v.3.6.0) (R: A Language and Environment for Statistical Computing, 2019). Alpha-diversity was calculated using phyloseq and Shannon (Spellerberg and Fedor, 2003), Simpson (1949), and Chao (1984) metrics. The diversity of samples was calculated using OTU-based Bray–Curtis dissimilarity and principal coordinates analysis (PCoA) as an ordination method and clustering with phyloseq. To normalize OTU abundance, the phylotypes that contained unclassified taxa were removed, and then data was normalized with Hellinger’s transformation (vegan package). A statistical comparison between the obtained values was performed using ANOVA and Tukey tests, with prior verification of data normality. For downstream analysis, samples were clustered using the kmeans function, based on the distance between the samples and separately based on a principal coordinate analysis. The number of clusters was determined using the fviz_nbclust function and the “silhouette” method. Taxonomic differences between samples and clusters were conducted using the STAMP software (Parks et al., 2014). The significance of the relative difference in proportion in the taxonomic distribution at the Family level of BT1 and BT2 samples was assessed using Fisher’s two-sided exact test, with the Newcombe-Wilson confidence interval method and Benjamin–Hochberg’s FDR being used for correction. Welch’s *t*-test, Welch’s inverse test for the confidence interval method, and Benjamin–Hochberg’s FDR for adjustment were used to compare clusters. The core microbiome was obtained using the R package microbiome and the plot_core function.

## Results

### Taxonomic Assignments of Obtained Isolates, Cultivated Under Aerobic Conditions

**FIGURE 1:**
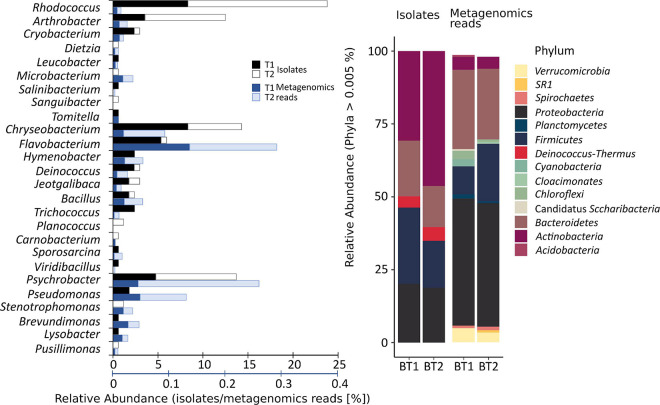
Comparison of the number of obtained isolates with the relative abundance of microorganisms present in the studied environment. The upper black axis relates to the relative abundance of isolates, while the lower axis relates to metagenomic reads. T1 and T2 refer to the time of sampling, 14.01.2012 and 02.02.2012, respectively, from which isolates and reads were obtained. The relative abundance was obtained based on the 16S rRNA gene fragment sequencing. The isolates constitute a small fraction of overall biodiversity and different taxonomic shifts between T1 and T2.

To obtain the widest possible diversity of cultivated strains under aerobic conditions, the water samples were spread onto plates with an oligotrophic, low-nutrient R2A medium. This medium allows for the culture of slow-growing bacteria that might be limited in full media or might be suppressed by faster-growing isolates. The analysis of the number of heterotrophic bacteria and their taxonomy revealed a preliminary estimation of whether significant changes in the environment occurred. These results showed a significant difference in the number of colony-forming units (cfu) in both samples (*p* < 0.05 in *t*-test). In the case of the sample taken in January (T1), 5.26 × 10^4^ (±2.21 × 10^3^) cfu mL^–1^ were obtained, and 1.22 × 10^6^ (±7.44 × 10^4^) cfu mL^–1^ were obtained in the case of the sample taken in February. According to the differences in color and morphology, 84 colonies from each sample were selected and passaged, and their taxonomic affiliation was determined as well. The identified bacteria were classified into five taxonomic phyla (Figure 1) represented by 28 different genera and assigned into 57 different phylotypes (34 at T1 and 33 at T2) and 33 species (20 in T1 and 16 unique species at T2), based on the similarity criteria of ≥99% and ≥97% of the 16S rRNA gene sequence, respectively (Supplementary Table 2). It was shown that *Actinobacteria* was the predominant phylum in each of the samples (T1 – 34%, T2 – 57%), followed by *Bacteroidetes* (32, 13%), *Proteobacteria* (15, 22%), and *Firmicutes* (14, 7%). The most frequently identified bacterial isolates belonged to the genera *Rhodococcus* (23.9% of isolates), *Chryseobacterium* (14.3%), *Psychrobacter* (13.7%), *Arthrobacter* (12.5%), and *Flavobacterium* (6%).

### Metabolic Properties of Obtained Isolates

**FIGURE 2:**
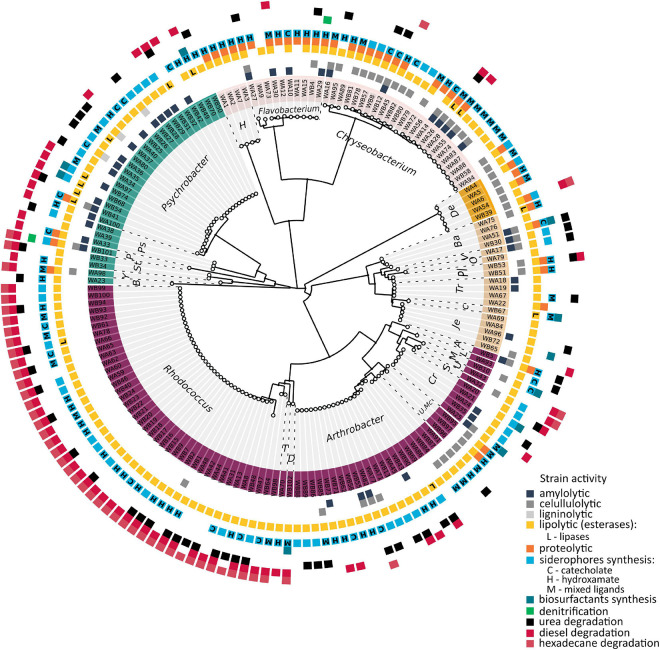
Summary of the metabolic properties of obtained bacteria, in association with their phylogenetic relationships. The evolutionary history was inferred by using the Maximum Likelihood method and General Time Reversible model. The tree with the highest log likelihood (–8347.03) is shown. Initial tree(s) for the heuristic search were obtained automatically by applying Neighbor-Join and BioNJ algorithms to a matrix of pairwise distances estimated using the Maximum Composite Likelihood (MCL) approach, and then selecting the topology with superior log likelihood value. A discrete Gamma distribution was used to model evolutionary rate differences among sites [four categories (+G, parameter = 0.5689)]. The rate variation model allowed for some sites to be evolutionarily invariable [(+I), 26.12% sites]. The analysis involved 168 nucleotide sequences. All positions containing gaps and missing data were eliminated (complete deletion option). There were a total of 827 positions in the final dataset. Evolutionary analyses were conducted in MEGA X. The labels W(A–B)(1–102) refer to the obtained isolates. Background color refers to Phyla: purple, *Actinobacteria*; light brown, *Firmicutes*; orange, *Deinococcus-Thermus*; pink, *Bacteroidetes*; and cyan, *Proteobacteria*. Abbreviated genus names mean: T, *Tomitella*; D, *Dietzia*; U. Mc, unclassified *Micrococcaceae*; Cr, *Cryobacterium*; S, *Salinibacterium*; U, unclassified *Microbacteriaceae*; M, *Microbacterium*; L, *Leucobacter*; A, *Sanguibacter*; Je, *Jeotgalibaca*; C, *Carnobacterium*; Tr, *Trichococcus*; Pl, *Planococcus*; O, *Sporosarcina*; V, *Viridibacillus*; Ba, *Bacillus*; De, *Deinococcus*; H, *Hymenobacter*; Ps, *Psychrobacter*; P, *Pseudomonas*; St, *Stenotrophomonas*; Y, *Lysobacter*; and B, *Brevundimonas*.

To investigate the function of the pond community, metabolic tests of the isolated bacteria were performed (Figure 2). These tests focused on the ability to (i) decompose complex organic compounds, (ii) synthesize selected secondary metabolites, and (iii) transform selected nitrogen compounds. Biochemical properties associated with the degradation of complex organic compounds that are potentially present in the pond and thus enable cycling of organic matter in the studied environment were analyze to evaluate the overall metabolic potential of microbial communities. The analysis indicated that lipolytic, cellulolytic, proteolytic, and amylolytic properties possess, 96, 30, 26, and 26% of the tested strains, respectively. Among the strains with lipolytic properties, 81% showed the ability to produce tributyrin esterases, and 15% to produce tributyrin esterases as well as lipases. Most strains possessing lipolytic activity belonged to *Actinobacteria* phylum – 55%. Among them, 65% constituted *Rhodococcus* and 57% *Arthrobacter* species. Most strains possessing proteolytic properties (63%) and cellulolytic properties (41%) belonged to *Bacteroidetes* – *Chryseobacterium* and *Flavobacterium*. In total, 58 and 90% of the isolates from the first and second genera, respectively, showed the ability of casein proteolysis, and 79 and 20% demonstrated the ability to degrade carboxymethylcellulose. Among the strains able to decompose starch, 42% belonged to *Proteobacteria*, of which 35% were *Psychrobacter* and 26% were *Bacteroidetes* – *Chryseobacterium* and *Flavobacterium* strains. Among the investigated strains, three *Psychrobacter* spp. were able to produce extracellular laccases.

The sampled environment is a pristine area, where no petroleum hydrocarbon contamination was found. Hence, we decided to assess whether the isolated strains possessed the ability to grow on these organic compounds and, thus, whether they might constitute a permanent component of the sampled pond. According to the performed analysis, it was confirmed that 44% of isolated bacteria showed the ability to grow on diesel and 29% to grow on n-hexadecane, including 98% of *Rhodococcus* and 24% of *Arthrobacter* strains growing on diesel, and 100 and 5% of these strains growing on hexadecane, respectively.

The next investigated metabolic properties concerned the synthesis of siderophores and biosurfactants. Iron is one of the key factors required for many cellular processes, including respiration, the lack of which limits the growth of most bacteria. To overcome iron limitation, bacteria produce siderophores to complex iron with an affinity for other trace metals as well. The ability to complex metals may be exhibited by biosurfactants, which, due to their amphiphilic structure, increases the bioavailability of organic compounds and may affect their mobilization. These two types of compounds not only affect the condition of producers but could have implications for the condition of other members of the community. In total, 87% of bacteria showed the ability to synthesize siderophores and 3% to produce biosurfactants. Most of the strains in the first group belonged to *Actinobacteria* and included 90% *Rhodococcus* spp. and 76% *Arthrobacter* spp. According to the performed Arnow’s and Snow’s tests, 13% of strains produced siderophores with the catechol group, 32% the hydroxamate group, and 22% both of these groups. Only five strains showed the ability to produce biosurfactants – *Psychrobacter* sp. ANT_WB68, ANT_WB70, and ANT_WB74, *Bacillus* sp. ANT_WA51 and *Dietzia* sp. ANT_WB102. Only *Bacillus* sp. ANT_WA51 demonstrated the ability to constitutively produce these metabolites. The remaining strains required induction with a hydrophobic substance (vegetable oil).

Due to the presence of nitrates in the sampled environment, the ability of the isolates to denitrify was also verified. Only two strains *Flavobacterium* sp. ANT_WA29 and *Pusillimonas* sp. ANT_WB101 were able to perform this process. In addition, the ability of the isolates to degrade urea was tested. Thirty-two percent of the strains showed ureolytic activity, including 53% of strains belonging to the genus *Rhodococcus*, 57% to *Arthrobacter*, and 26% to *Psychrobacter*.

### Structure of Total Bacterial Populations and Their Changes

To determine what part of the bacterial community was successfully isolated and whether it was a representative group of the community, the high-throughput sequencing of a 16S rRNA gene fragment was performed (Figure 1).

The obtained data shows that the taxonomic structure of the total bacterial community is different from the obtained isolates. *Proteobacteria* (T1 – 42.30%, T2 – 41.21% of relative abundance), *Bacteroidetes* (26.39, 23.51%), *Firmicutes* (9.32, 19.18%), *Verrucomicrobia* (4.71, 3.25%), *Chloroflexi* (2.72, 0.82%), *Actinobacteria* (4.28, 3.99%) and *Cyanobacteria* (2.38, 0.33%), *Planctomycetes* (0.55, 1.00%), and *Spirochaetes* (1.14, 0.99%) dominated. The rest of the bacterial phyla (34) constituted low percentages (<1%). Among the classified genera, *Tychonema* (1.74, 0.02%) from *Cyanobacteria*, *Luteolibacter* representing *Verrucomicrobia* (1.21, 1.18%), *Flavobacterium* (0.79, 0.97%) and *Persicitalea* (0.21, 1.38%) representing *Bacteroidetes*, *Psychrobacter* (0.26, 1.35%) belonging to *Proteobacteria* and *Tissierella* (0.37, 0.76%) from *Firmicutes* contributed to the highest proportion. In contrast, amongst cultivable bacteria the most abundant were *Flavobacterium* and *Psychrobacter*. *Rhodococcus*, the most abundant isolate, constituting 23%, represented less than 0.05% of biodiversity inspected via metabarcoding (T1 – 0.46%, T2 – 0.38%).

To examine whether the different bacterial community structures in the sample collected in January and February could have resulted from penguin and sea elephant effluent runoff, we analyzed the nitrogen and phosphorus content of the samples. The analysis showed a significant increase in these elements. Comparing the T2 sample with the T1 sample, the concentration of ammonium ions, nitrates, and phosphorus increased about twofold (Supplementary Table 1). A more than twofold decrease in sulfate content was also found.

By examining the changes in the relative abundance of particular taxa along the time course it was revealed that at the family level, excluding unclassified taxa, the greatest decrease in abundance (>1%, *p* < 0.05) was observed for *Microcoleaceae* of *Cyanobacteria* phylum, *Flavobacteriaceae* and *Cyclobacteriaceae* of *Bacteroidetes*, *Anaerolineaceae* of *Chloroflexi*, *Verrucomicrobiaceae* of *Verrucomicrobia*, *Helicobacteraceae*, *Alteromonadaceae*, and *Desulfobacteraceae* of *Proteobacteria*. The greatest increases (>1%, *p* < 0.05) were observed for *Comamonadaceae*, *Moraxellaceae*, and *Sphingomonadaceae* of *Proteobacteria*, *Porphyromonadaceae*, *Cytophagaceae* of *Bacteroidetes*, and *Ruminococcaceae* of *Firmicutes*. Among the groups of photosynthetic bacteria, a slight change in abundance also occurred. An increase in the number of purple sulfur bacteria – *Chromatiaceae* (from 0.33 to 0.92%, *p* = 0.00), purple non-sulfur bacteria – *Rhodospirillaceae* (0.42 to 0.44%, *p* > 0.23), and green sulfur bacteria – *Chlorobiaceae* (0.022 to 0.098%, *p* = 0.08) were observed. The species included in the ammonia-oxidizing bacteria (AOB) – *Nitrosomonas*, *Nitrosococcus*, and *Nitrosospira* as well as anommox – *Candidatus* Brocadia, and *Candidatus* Scalindua – were found to reduce its amount. A decrease in the amount of δ*-Proteobacteria* was also noted, the representatives of which belong to the *Desulfobacterales*, *Desulfovibrionales*, and *Syntrophobacterales* groups of sulfate-reducing bacteria (SRB). In addition, at T1, among the *Cyanobacteria*, microbes with the ability to fix nitrogen were identified. These included the genera filamentous, non-heterocystous and unicellular *Oscillatoria* (3.61% of all *Cyanobacteria*), *Gloeothece* (0.05%), and *Lyngbya* (0.02%), and filamentous, heterocystous *Brasilonema* (0.09%).

To investigate whether there could have been changes in the metabolism of the studied bacterial communities, a prediction of their metabolic properties based on the 16S rRNA gene sequences and the potential of the strains annotated in the KEGG database was performed. A summary of enzyme abundance and the contribution of each metabolic pathway is presented in Supplementary Table 3. The greatest differences (>10% of abundance) occurred for the following metabolic pathways: chemical structure transformation maps (26%), xenobiotics biodegradation and metabolism (25%), glycan biosynthesis and metabolism (15%), and biosynthesis of other secondary metabolites (11%), followed by nucleotide metabolism (9%), metabolisms of other amino acids (6%), and lipid metabolism (6%). Of the enzyme activities, the highest abundance was found for proteases (caseinolytic; 1E-03), amylases (alpha-amylase and isoamylase; 3E-04), lipases (putative tributyrin esterase and triacylglycerol lipase; 1E-04), and cellulases (cellulose 1,4-beta-cellobiosidase and endoglucanase; 4E-05). In addition, cellulases increased in abundance by 29% and lipases by 57%, moreover amylases decreased in abundance by 7% and proteases by 12%. An increase in abundance occurred for enzymes involved in pathways related to nitrogen metabolism. In the case of the pathway: (1) denitrification – 14%, (2) nitrification – 47%, and (3) conversion of uric acid, through urea to ammonia – 32%. An increase in abundance was also observed for enzymes involved in phosphorus metabolism (on average by 18%). Among the obtained results, a considerable increase in the abundance of proteins responsible for arsenite metabolism was also observed – arsenite oxidase >200%.

### Biodiversity of Antarctic Bacteria in Relation to the Analyzed Water Body

In order to determine the most similar environment to the sampled pond as well as their taxonomic similarity with communities in other Antarctic environments, 16S rDNA amplicons of seawater, lake water, pond, soil, sand rock, deep marine sediment, and feces samples were analyzed (Supplementary Table 4). To compare the communities found in different environments versus the pond, at first, we started the analysis by grouping the samples according to the (dis)similarity between them (cluster number) and the source environment (cluster prefix).

**FIGURE 3:**
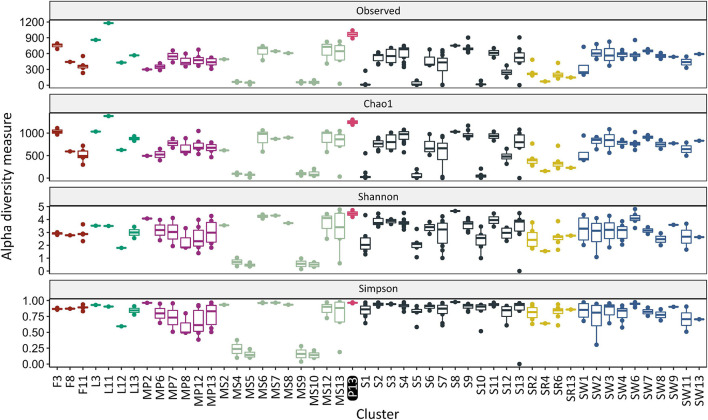
Alpha diversity occurring in various Antarctic environments in relation to the study pond (Cluster P13). Different colors indicate different sources of isolation. The cluster prefix refers to the isolation source: F, feces; L, lake; MP, meltwater pond; MS, marine sediment; P, pond from this work; S, soil; SR, sandstone rock; and SW, seawater.

The obtained results indicated that the richness, expressed as number of observed OTUs (Sobs), was the greatest for the sample from water columns of McMurdo Dry Valley Lake – 1179 (cluster L11) and the samples analyzed in this study – average 964.5 ± 106.8. The Sobs values for other clusters were below 900, and for most clusters significantly lower (*p* < 0.05). To estimate the richness at the genus level, the Chao1 index was used. The greatest value was found for the cluster L11 – 1378 and the cluster of water pond samples analyzed in this study – 1,242 and was significantly different from three out of four clusters of seawater samples (SW6/8/10), five out of 13 soil clusters (S1/5/7/10/12), four out of 10 sediment clusters (MS 4/5/9/10), the feces cluster (F11), and all sand rock clusters. The number of observed OTUs for samples of the pond constituted 78% of the estimated richness value, whereas, for samples of lake, feces, sediment, seawater, soil, and sand rock, it constituted about 75, 73, 73, 73, 67, and 59% of the value. To measure microbiological diversity, (inverted) Simpson (HI) and Shannon (DI) indices were used. These indices estimate the richness and evenness of the genera, although the first is more concerned with genus evenness, while the second is more concerned with richness. The greatest diversity was observed in the soil sample (cluster S8, HI – 0.98, DI – 4.65), ponds (HI – 0.97, DI – 4.46), and sediment (MS7, HI – 0.97, DI – 4.30). Significantly different DI and HI values were found between the pond cluster and sediment (MS4/5/9/10). The alpha-diversities are shown in Figure 3 and summarized in Supplementary Table 5.

**FIGURE 4:**
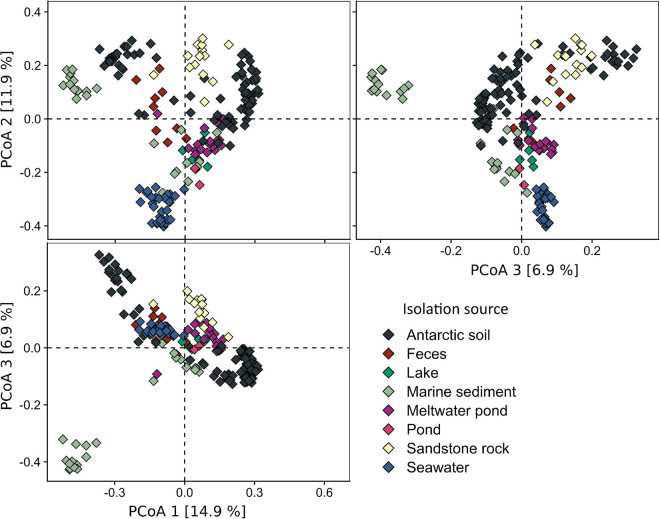
Beta diversity of Antarctic environments shown as principal coordinate analysis (PCoA) based on the Bray–Curtis dissimilarity matrix.

**FIGURE 5:**
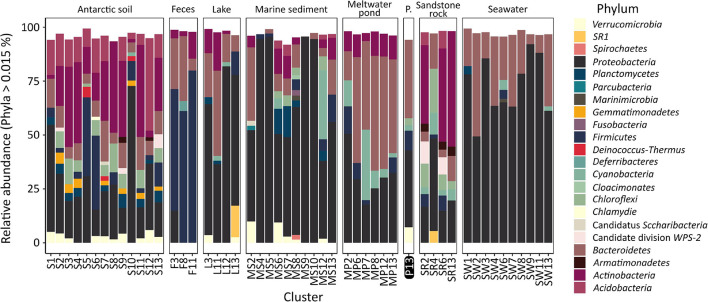
Taxonomic diversity of clusters of analyzed samples at the phylum level. P., the pond analyzed in this study.

The analysis of the diversity between each of the samples (beta-diversity, Figure 4), carried out using PCoA and hierarchical clustering based on Bray–Curtis metrics, showed that the samples from the pond are most similar to lake (cluster L11: dissimilarity – 0.55 ± 0.02), meltwater pond (MP7: 0.59 ± 0.01, MP12: 0.59 ± 0.04), seawater (SW7: 0.59 ± 0.03), marine sediment (MS2: 0.59 ± 0.02), soil (S8: 0.59 ± 0.01), and penguin guano samples (F3: 0.60 ± 0.04). The lowest dissimilarity occurred between sample T1 and the lake L4 sample, followed by seawater samples (SW8/4/9/32/6/1/3), and, in case of sample T2, with meltwater pond (MP3/16/20) and feces (penguin guano samples). The dissimilarity between the pond samples was 0.43. Taxonomic analysis at the phylum level (Figure 5) showed that *Proteobacteria* (dominated in the all lake, meltwater pond, marine sediment, and seawater clusters), *Bacteroidetes*, *Actinobacteria* (in S3, SR2/6/13), *Firmicutes* (in the feces clusters), *Acidobacteria*, *Planctomycetes*, *Chloroflexi*, *Verrucomicrobiota*, *Cyanobacteria*, and *Gemmatimonadetes* were the most abundant taxa among all clusters. The rest of the phyla included 39 different taxa. The main difference (*p* < 0. 05) at this taxonomic level with the pond was found in the following clusters: feces F11 (*Firmicutes* and *Proteobacteria*), melted pond MP11 (*Actinobacteria*), marine sediment MS5/10 (*Proteobacteria* and *Nitrospirae*), soil S2 (*Actinobacteria*), soils S4/9 (mainly *Actinobacteria*, *Proteobacteria*, *Acidobacteria*, and *Planctomycetes*), sand rocks SR2 (*Actinobacteria* and *Proteobacteria*), SR6 (*Proteobacteria*), and seawater SW4 (*Proteobacteria* and *Actinobacteria*). The taxonomic characteristics of the bacterial community performed at the family level revealed that out of 347 classified taxa, one (*Moraxellaceae*) was shared by all clusters (none by all samples), and with a minimum threshold of 0.1% of relative abundance and 0.9 of prevalence, the amount was 18. The greatest prevalence was observed for bacteria belonging to *Moraxellaceae* and *Pseudomonadaceae* (*Proteobacteria*), *Flavobacteriaceae* (*Bacteroidetes*), and *Planctomycetaceae* (*Planctomycetes*). However, considering the data only for communities grouped with a pond (cluster 13), the number of shared families within samples was 10, with the above threshold criteria. In this case, the greatest prevalence occurred for *Pseudomonadaceae*, *Enterobacteriaceae*, *Moraxellaceae* and *Rhodobacteraceae* (*Proteobacteria*), *Flavobacteriaceae* and *Chitinophagaceae* (*Bacteroidetes*), and *Planctomycetaceae* (*Planctomycetes*). At the genus level, no shared taxon was observed for all samples or clusters. Among 2,070 classified genera, all occurred in environments of cluster 13. The most common genera were *Psychrobacter* and *Pseudomonas* from *Proteobacteria*, *Flavobacterium* from *Bacteroidetes*, *Ilumatobacter* from *Actinobacteria*, and *Gemmatimonas* from *Gemmatimonadetes*. In the pond, there were also genera not detected in other analyzed environments with low abundance (>0.015%): *Catenococcus*, *Macromonas*, (*Proteobacteria*), and *Mongoliicoccus* (*Bacteroidetes*).

## Discussion

Polar environments constitute approximately 15% of the Earth’s biosphere. One of them is the Antarctic realm, the coldest, driest and windiest, and, thus, most difficult place to live on Earth. Although the number of studies reporting on microbial communities and their bioprospecting has been growing almost exponentially, our knowledge about the ecology and diversity of Antarctic environments remains limited. We know relatively little about the functioning of aquatic ecosystems, which consist of marine, freshwater, lotic, wetlands, and lentic ecosystems. Among the lentic ecosystems, the most studied are lakes while less attention is paid to ponds, which can have a significant impact on the carbon and nitrogen cycles in polar environments. Furthermore, as recent studies show, High Arctic polygonal landscapes can also contribute to methane emissions (Kuhn et al., 2018). Given this lack of knowledge, we decided to assess the ecology of a pond located on King George Island.

The investigated environment, due to extreme weather conditions and glaciers, is a hard-to-reach place and is located on volcanic and sedimentary rocks (Birkenmajer, 2003) surrounded by the waters of the Bransfield Straits. It is also affected by wildlife – mainly penguins and seals – as well as lichens, grasses, and mosses. Therefore, this research helps to provide a better understanding of the complexity and functioning of bacteria communities found in such environments and of the processes that occur within them. The months during which samples were collected – January and February – are the warmest, which encourages greater wildlife and microorganism activity. In the study pond, a more than twofold increase in heterotrophic bacterial cell abundance, able to grow in the aerobic laboratory conditions, was observed between samples collected during this period.

Through a comparative analysis of the 16S rRNA gene sequence, we identified that *Actinobacteria* (*Rhodococcus* and *Arthrobacter*), *Bacteroidetes* (*Chryseobacterium* and *Flavobacterium*), *Proteobacteria* (*Psychrobacter*), and *Firmicutes* (*Bacillus*) are the dominant phyla. The number of isolates represented the remaining *Deinococcus*; *Thermus* phylum was the least. The identified taxa represented the most common phyla of heterotrophic bacteria detected by culture-based methods in polar environments (Peeters et al., 2011, 2012; Zdanowski et al., 2013; González-Rocha et al., 2017). Although there was an increase in the number of heterotrophic bacteria, as shown by the analysis of the number of colony-forming units of bacteria, there was a decrease in their diversity, which is also reflected in the decrease in richness and diversity of the total bacterial community.

Antarctic spring and summer are the time when penguins arrive on King George Island to breed (Gryz et al., 2018), as well as elephant seals (Fudala and Bialik, 2020). In places where colonies of these animals aggregate, their excrement enriches the soil and aquatic ecosystems with nitrogen and phosphorus compounds. As the results of chemical analyses indicate, in the sampled pond, there was a significant increase in nitrogen and phosphorus content, which contributed to pond eutrophication. The total concentration of nitrogen and phosphorus significantly exceeded the average value obtained for small and shallow water bodies and wetland areas around King George Island (Neogonekdzarek and Pociecha, 2010). In the investigated environment, there were 94–129 mg/L of total nitrogen and 20.02–42.08 mg/L of total phosphorus, while for the compared waters, there were 0.176–29.21 mg/L and 18.35 mg/L, respectively. The highest concentrations were achieved for reservoirs exposed to penguins. The increase in nitrogen and phosphorus concentrations contributes to enhancing the growth of mosses, lichens, as well as invertebrate and microbial communities, which, in addition, can utilize organic matter provided by the animals. Thus, having knowledge on the taxonomy of the isolates obtained, their metabolic potential was tested in order to determine the role of the isolates in the pond functioning. The conducted tests indicated that majority of isolated bacteria are able to degrade triglycerides and cellulose, followed by proteins, starch, and lignins. The lipolytic properties of bacteria may result from the activity of two main classes of hydrolase enzymes – esterases (EC 3.1.1.x) and lipases (EC 3.1.1.3) – and these two classes of enzymes were tested. According to analyses, only 15% of strains (mainly, strains belonging to *Psychrobacter*) were able to produce lipases and the remaining 81% produce esterases unable to hydrolyze esters with long-chain fatty acids. Cellulose, an essential component of cell walls, lignin, the second most abundant polymer from biomass, and starch, the most important plant energy storage, are the most common biopolymers in many environments. Degradation of these compounds in nutrient-poor environments may well confer an advantage to a microorganism able to perform these processes. The ability to produce extracellular laccases that degrade lignins, a highly branched phenolic polymer, was demonstrated by only three strains of *Psychrobacter*, which, however, are not capable of cellulose degradation. Investigated strains able to decompose cellulose and starch and that showed proteolytic properties belonged mainly to *Bacteroidetes* – *Chryseobacterium* and *Flavobacterium*.

Although the studied pond belonged to a pristine environment where no contamination with petroleum compounds had been observed, we decided to examine whether the gene pool formed in this pond contained those that enable microorganisms to utilize such compounds as a source of carbon and energy. According to the obtained results, 44% of bacteria were able to grow on diesel fuel and 29% on n-hexadecane. Of these, all isolates obtained from the genus *Rhodococcus* were able to grow on acyclic hydrocarbons and representatives of *Arthrobacter* in particular. The higher number of strains able to grow on diesel is probably due to the fact that diesel fuel is a mixture of hydrocarbons with different chain lengths and contains an addition of esters. The occurrence of bacteria capable to degradate aliphatic hydrocarbons may also be the result of metabolic evolution. In aquatic environments, a natural source of alkanes may be *Cyanobacteria*, of which anabolic activity is probably sufficient to sustain populations of hydrocarbon-degrading bacteria (Lea-Smith et al., 2015).

Among the tested strains able to grow on hydrocarbons was *Dietzia*, which, as well as *Bacillus* and *Psychrobacter* strains, possess the ability to improve the bioavailability of organic matter by producing biosurfactants. For most of these strains, except *Bacillus* sp. ANT_WA51 induction with a hydrophobic compound was necessary. In this work, we tested only vegetable oil, n-hexadecane, and diesel; however, cyclic hydrocarbons as well as various fatty acids can also act as inductors (Pal et al., 2009; Niu et al., 2019).

Due to the critical importance of iron in cell function, we examined the ability of isolates to synthesize siderophores. The conducted study showed that most isolates are capable of synthesizing siderophores, which have different characteristic functional groups: hydroxyl-, catechol-, or mixed. The wide variety of siderophores may be conducive to binding not only iron cations but also di, tri-, and tetravalent metal cations. Different bacterial requirements for iron and trace elements may be involved in a competition among bacteria, including cyanobacteria, and may also be related to taxonomic shifts. In addition, various type of siderophores may mobilized metals from the rocks, contributing to the circulation of metals in nature.

As the final tested metabolic property, we included the ability to degrade urea formed during uric acid decomposition and the ability to denitrify. Most of the identified ureolytic strains belong to *Actinobacteria* (*Rhodococcus* and *Arthrobacter*) and *Proteobacteria* (*Psychrobacter*). These bacteria, through the decomposition of urea and formation of ammonia, may cause the alkalization of the environment and, thus, the precipitation of minerals playing a fundamental role, i.e., in the calcium biogeochemical cycle.

The above analyses were performed only under laboratory conditions, which may be inappropriate to obtain the expression of genes involved in the particular metabolic pathways. One of the important factors here may be the change in ambient temperature. The average temperature in the warmest months in King George Island does not exceed 10°C (Araźny et al., 2013), while cultures were conducted at 20°C. However, the results indicate a wide metabolic potential of the obtained isolates, which may degrade either the compounds included in the biomass of phototrophs or provided by the fauna in the form of feces.

Moreover, in the pond, there were bacteria able to decompose alkanes, which indicates this environment may be able to self-purification. In general, these results support the observation that the hydrolytic activity of complex organic compounds is widespread among microorganisms, especially lipolytic activity (Ghosh et al., 1996). As indicated by the authors (Ferrés et al., 2015), this property might be more evident in microorganisms found in oligotrophic environments, where carbon is fixed by phytoplankton mainly into lipids or proteins (Henderson et al., 1991). Our study shows that there is no correlation between phenotypic features and phylotypes, indicating high metabolic diversity within species. Similar results were obtained by studying cryoconite hole microorganisms (Poniecka et al., 2020). Although some microorganisms were closely related, they showed striking differences in metabolic abilities. This may be due to the fact that freshwater habitats act as a bridge for gene exchange between differently disjunct habitats, and the gene pool is shaped by their broad ecological niche (Fondi et al., 2016).

To obtain a broader view of the pond processes and to determine the ecological role of the bacterial communities present at this site, predictions and examination of functional profiles were performed using Tax4Fun2. The obtained data indicated the dominance of pathways related to metabolism of carbohydrate (8.73%) and amino acids (7.67%), signal transduction (5.32%) and membrane transport (4.93%) followed by energy metabolism (3.88%), metabolism of cofactor and vitamin (3.32%), lipid metabolism (3.18%), xenobiotics biodegradation and metabolism (2.92%), and metabolism of nucleotide (2.03%). These results, as well as previous studies of bacterial communities in aquatic ecosystems, included eutrophic ones, indicate a reliance of bacterial metabolism on synthesis and degradation processes of organic compounds (Zhou et al., 2018; Zhang et al., 2020; Papale et al., 2021). Nonetheless, the most dominant pathways were metabolism of amino acid and membrane transport. This difference may be related to the availability of different nutrients as well as the form of occurrence of the microorganisms – free-living versus particle-associated (Jankowiak and Gobler, 2020). Over time, it was noted that there was an increase in the proportion of pathways associated with metabolism of propionate, butyrate, glyoxylate and dicarboxylate, pyruvate and lipid, and benzoate degradation. The observed proportions of metabolic pathways indicated that the observed bloom has not yet reached the decline stage (Zhou et al., 2018) and the similarity in functional properties of the bacteria despite taxonomic differences implies a high level of occurrence of genes performing the same function and less functional diversity. The performed prediction of metabolic potential as well revealed consistency in the tendency of prevalence of enzymes related to the degradation of complex organic compounds, with the data obtained from the analysis of metabolic potential of isolates. In both cases, there was a decrease in abundance of amylases and proteases, as well as an increase in abundance of lipases and cellulases. It should be noted that tools which predict metabolic potential based on 16S rRNA gene sequences only provide an estimate of possible gene content based on a reference genome database. Thus, further functional studies based for example on metagenomics and metabolomics are needed to determine the exact role of bacterial communities in the studied environments.

After isolation and examination of the metabolic potential of heterotrophic bacteria, it was verified whether the obtained bacteria were a group representative of the total community. The obtained results indicated that the biodiversity of the pool of cultivable bacteria represented a small percentage of the total microbial diversity in the environment. Analysis of bacterial communities indicated that the taxa *Tychonema*, *Luteolibacter*, *Persicitalea*, *Petrimonas*, and *Tissierella* of which no representatives were obtained under laboratory conditions, showed the highest relative abundance, as well as *Flavobacterium* and *Psychrobacter* – genera that were also identified among the isolates. *Tychonema* is a genus of filamentous *Cyanobacteria*, that is unable to fix nitrogen. It was identified in e.g., microbiome in Perennially Ice-Covered Lake Untersee in East-Antarctic (Weisleitner et al., 2019), cryoconite hole in Untersee Oasis (Weisleitner et al., 2020), Biscoe and Elephant Point in Soils From the Antarctic Peninsula Region (Almela et al., 2021), where it represented the most abundant *Cyanobacteria. Luteolibacter* represents the phylum *Verrumicrobia* – a group of microorganisms found in a wide range of habitats, most of which are eutrophic (Schlesner et al., 2006), *Persicitalea*, *Petrimonas*, and *Flavobacterium* represent *Bacteroidetes* which play an important role in the degradation of organic compounds, including insoluble polymeric substrates (Thomas et al., 2011), whereas *Firmicutes* represented by *Tissierella* are involved in the degradation of biopolymers (Mahowald et al., 2009; Flint et al., 2012), and *Psychrobacter* represents *Proteobacteria*, which frequent occurrence is thought to reflect their adaptability to environmental changes, including extreme environments (Edwards et al., 2014). High abundances of individual groups of heterotrophic bacteria were described previously as a result of high grazing pressure (Bianchi, 1989). Moreover, phytoplankton bloom may have contributed to favoring bacteria of the genus *Luteolibacter*, *Flavobacterium* and bacteria belonging to the family of *Cytophagaceae* (no data available for *Persicitalea*), which possess the ability to degrade algal and cyanobacterial products and cause their lysis (Mayali and Azam, 2004; Ohshiro et al., 2012). Additionally, the presence of animals may have contributed to the increased abundance of *Psychrobacter*, *Tissierella*, and *Petrimonas*. *Psychrobacter* is common in ornithogenic soils derived from bird guano and may also be found in their digestive system as well as in krill (Denner et al., 2001; Yew et al., 2018; Grzesiak et al., 2020). Bacteria of the genus *Tissierella* and family *Tissierellaceae* are also found in feces of penguins (Guo et al., 2018; Grzesiak et al., 2020), while *Petrimonas* are found in feces of southern elephant seals and leopard seals (Nelson, 2012). In contrast to culture-based analyses, *Rhodococcus* and *Arthrobacter* represent a slight proportion of relative abundance in metagenomic analyses. The structure of the total bacterial communities differs from that of the isolates. The dominant phyla are *Bacteroidetes, Proteobacteria, Firmicutes, Chloroflexi*, and *Cyanobacteria*. These results appear to be structured similarly at the phylum level with previous works (Archer et al., 2015). As indicated by the Shannon and Simpson indices, the diversity at the genus level is lower for cultured strains compared to the total microbiome (1.8 times higher and 4 times lower indices, respectively).

Previous studies carried out on glacier cores from the Tibetan Plateau (Liu et al., 2019) indicated that incubation temperature was an important factor influencing isolate communities. Conducting cultures at different temperatures resulted in a diverse pool of isolates, with the greatest diversity achieved at low temperature (Christner, 2002; Liu et al., 2019). Studies of bacterial communities in soil at the forefield of the subarctic glacier (Mateos-Rivera et al., 2016) indicated the dependence of microbial diversity on soil surface temperature as well. In contrast, studies on the effects of abiotic factors on the marine bacterial communities of the Potter Cove (Hernández et al., 2019) and communities of the Dry Valleys (Antarctic) soils (Bottos et al., 2020), showed that temperature exerted the weakest direct influence on the composition of the communities. These results suggest that a change in abiotic factors will promote the growth of certain groups of microorganisms, and in order to be able to reliably compare the structures of cultivated and non-cultivated communities, microbial diversity needs to be investigated at a lower ambient temperature (e.g., 4°C).

Studies on the physicochemical changes and the taxonomic structure of the bacterial communities revealed that progressive eutrophication resulted in a decrease in the richness and diversity of bacterial communities at the genus level – the Chao and Shannon indices decreased by 1.15 and 1.02-fold, respectively, and the Simpson index increased by 1.24-fold. The decrease in biodiversity may be related to increased nutrient concentrations, of which previous studies indicate may be the cause of reduced richness in aquatic bacterial communities (Drury et al., 2013; Zhang et al., 2020). The highest abundance changes were found for *Firmicutes* – a threefold increment, a threefold decrement of *Chloroflexi* to below 1%, and a sevenfold decrement of *Cyanobacteria*. *Clostridia* showed the greatest increase among *Firmicutes* (about a twofold increment to 15% of relative abundance), including *Ruminococcaceae* (found in a high proportion in seal feces), *Clostridiales* Incertae Sedis XI, XII, and XII (mainly in penguin feces) and *Acidaminococcaceae* (in seal feces). The increase in abundance of these bacteria, as well as, the decrease in relative abundance of anaerobes, *Anaerolineaceae* of *Chloroflexi*, may be a result of higher stress caused by higher solids, ammonia, and VFAs concentration (Hao et al., 2016) resulted from the continuous income of penguin sewage and seals into the pond. These results indicate that the bacteria belonging to *Clostridia* may be involved in the decomposition of decayed autotrophic biomass (Zhao et al., 2017), and together with members of *Bacteroidetes*, play a dominant role in the degradation of high molecular mass organic matter. *Cyanobacteria* are the dominant phototrophs in Antarctic freshwater and terrestrial habitats, considered to be the main primary producers responsible for increasing nutrient concentration through nitrogen fixation. In the analyzed system the reduced relative abundance of *Cyanobacteria* was observed. We speculate that between the samplings biological production was increased, oxygen content decreased, and excessive accumulation of cyanobacteria resulted in a reduction in their abundance. In addition to a decrease in oxygenic photosynthetic bacteria, there was a slight increase in the number of anoxygenic photosynthetic bacteria, including purple sulfuric bacteria – *Chromatiaceae*, purple non-sulfuric bacteria – *Rhodospirillaceae*, and green sulfuric bacteria – *Chlorobiaceae*.

In order to evaluate the biodiversity of different Antarctic environments and determine the similarity of the pond with other such environments, as well as the potential source of microorganisms, an analysis of an additional 192 amplicons was performed. The results indicate that the biodiversity in the pond samples was greater than that of most samples of seawater, soil, sand rock, feces, and other meltwater ponds and was similar to the diversity found in marine sediment and lake samples. The most similar bacterial communities, based on the Bray–Curtis metric, were the pond and lake located at McMurdo Dry Valleys – a site of largely snow-free valleys in Antarctica, located within Victoria Land, the seawater and meltwater pond samples in T1, and the pond and penguin fecal samples in T2.

Comparative analysis between the pond communities (T1 and T2) and the other meltwater ponds at the family level showed no statistically significant differences and indicated that in T1 there is a higher abundance of *Rhodobacteraceae* (*Proteobacteria*) and in T2 higher abundance of *Micrococcaceae*, *Microbacteriaceae* and *Propionibacteriaceae* (*Actinobacteria*), and *Propionibacteriaceae* (*Proteobacteria*). Taxonomic analysis conducted at the phylum level showed no statistically significant differences between samples from the analyzed pond and the most similar samples from penguin guano, lake, marine sediment, soil, and sand rock, in which the most common families were *Flavobacteriaceae* and *Chitinophagaceae* (*Bacteroidetes*), *Comamonadaceae*, *Xanthomonadaceae*, and *Rhodobacteraceae* (*Proteobacteria*). By contrast, considering all the analyzed samples, *Planctomycetes*, *Flavobacteriaceae*, and *Pseudomonadaceae* predominated. The performed comparative analyses, although they provide insight into the biodiversity of the pond and indicate the similarities between different Antarctic environments, are still incomplete. The challenge remains in conducting extensive comparative analyses of microbial communities, including Archaea and Fungi at the species level, which will not only allow us to determine the difference in community structures but also the dependence of their geographical distribution.

## Conclusion

In this study, we evaluated the biodiversity of bacterial communities present in a periodic pond located at King George Island (Antarctica) and assessed their metabolic potential, providing new insight into the ecology and functioning of habitats of this kind. The results suggest that this is a habitat with a diverse and rich microbial community potentially involved in (i) primary production – represented by oxygenic and anoxygenic photosynthetic bacteria, (ii) decomposition of organic matter – mostly represented by *Bacteroidetes* and *Firmicutes* – and (iii) nitrogen cycling – represented by ureolytic and denitrifying bacteria. The diversity of this type of habitat is strongly influenced by wildlife, mainly birds, which supply nitrogen and phosphorus compounds and enrich the indigenous microbiome. As a consequence of the succession, such waterbodies may evolve into biocenoses with favorable conditions for the growth of mosses, lichens, and grasses. Furthermore, our study indicates that in addition to the considerable taxonomic biodiversity, there is great variability in the biochemical properties of bacteria that may find applications in biotechnology.

## Data Availability Statement

The datasets presented in this study can be found in online repositories. The names of the repository/repositories and accession number(s) can be found in the article/Supplementary Material.

## Author Contributions

TK, LDr, and LDz conceived the study and wrote – review and edited the manuscript. TK performed all analyses, interpreted the data, and wrote the original draft. LDr and LDz consulted the results. LDz funded the acquisition. LDr supervised the study. All authors contributed to the final version of the manuscript.

## Conflict of Interest

The authors declare that the research was conducted in the absence of any commercial or financial relationships that could be construed as a potential conflict of interest.

## Publisher’s Note

All claims expressed in this article are solely those of the authors and do not necessarily represent those of their affiliated organizations, or those of the publisher, the editors and the reviewers. Any product that may be evaluated in this article, or claim that may be made by its manufacturer, is not guaranteed or endorsed by the publisher.

## Footnotes

1
http://www.digitalworldbiology.com/finchtv

2
https://www.ncbi.nlm.nih.gov/refseq/targetedloci/16S_process/

## Supplementary Material

Supplementary Table 1 Information about samples collected from the studied pond.

Supplementary Table 2 Taxonomy information about strains obtained from the studied pond.

Supplementary Table 3 Enzyme and metabolic pathway abundance occurring in the pond.

Supplementary Table 4 Summary information on the amplicons used for the pond comparison.

Supplementary Table 5 Alpha-diversity of the samples compared in this study.

Supplementary File 1 Extended description of the used methods.
