# Supplementary material for: Insight Into Ecology, Metabolic Potential, and the Taxonomic Composition of Bacterial Communities in the Periodic Water Pond on King George Island (Antarctica)
Source: Front Microbiol. 2021 Oct 8;12:708607. doi: 10.3389/fmicb.2021.708607 (PMC8531505; doi:10.3389/fmicb.2021.708607)
Supplement: Supplementary Table 1 — Information about samples collected from the studied pond. [file Table_1.docx]

Table S1. Information about samples collected from the studied Pond. Total nitrogen, nitrates, ammonium, total phosphorus, phosphates, sulfates and chlorides were measured spectrophotometrically using NANOCOLOR® kits and Photometer PF-12 (Macherey-Nagel GmbH & Co.), according to the manufacturer’s instructions. Urease (from *Canavalia ensiformis* seeds) used to determine urea content was obtained from Merck Millipore as powder with protein content of 26.9% and activity 346.0 U/mg. Urea was determinad directly, by convert difference in ammonia content of samples before and after enzyme treatment to urea based on the equation of reaction: (NH_2_)_2_CO + 2H_2_O → 2NH_4_^+^ + CO_3_^2-^. The reaction of conversion was performed by incubating 10 mL of sample with urease at a final concentration of 100 µg mL^-1^ at 20°C for 1 hour. pH and conductivity were measured using a pH/conductivity meter CPC-411 (Elmetron) and electrodes EPS-1 and ECF1 (Elmetron), respectively.

| **Parameters** | **Sample** | |
| --- | --- | --- |
|  | **T1** | **T2** |
| Total N [mg L^-1^] | 94±2 | 129±6 |
| NH_4_^+^ [mg L^-1^] | 42±1 | 77±2 |
| NO_3_^-^ [mg L^-1^] | 8±1 | 17±1 |
| Total P [mg L^-1^] | 20.02±2 | 42.08±1 |
| PO^4-^ [mg L^-1^] | 36±1 | 80±1 |
| SO_4_^2-^ [mg L^-1^] | 70±3 | 31±1 |
| Cl^-^ [mg L^-1^] | 103 | 420 |
| Urea [mg L^-1^] | 2.65 | 5.29 |
| pH | 7.9 | 8.4 |
| Conductivity [mS cm^-1^] | 0.415 | 1.848 |
