# Supplementary material for: Insight Into Ecology, Metabolic Potential, and the Taxonomic Composition of Bacterial Communities in the Periodic Water Pond on King George Island (Antarctica)
Source: Front Microbiol. 2021 Oct 8;12:708607. doi: 10.3389/fmicb.2021.708607 (PMC8531505; doi:10.3389/fmicb.2021.708607)
Supplement: Supplementary Table 2 — Taxonomy information about strains obtained from the studied pond. [file Table_2.docx]

Table S2 Taxonomy information about strains obtained from the studied Pond.

| *Phylum* | Genus (RDP classifier) | Strains | NCBI best hit | | | |
| --- | --- | --- | --- | --- | --- | --- |
|  |  |  | **Species** | **Coverage (%)** | **Identity (%)** | **Accession** |
| *Actinobacteria* | *Arthrobacter* | ANT_WB13; ANT_WB55; ANT_WB85; ANT_WB88 | *Arthrobacter alpinus* strain S6-3 | 100 | 98.39 | NR_117254.1 |
|  |  | ANT_WB90 |  | 100 | 98.51 |  |
|  |  | ANT_WB43; ANT_WA77; ANT_WA82 |  | 99 | 98.66 |  |
|  |  | ANT_WB89 | *Arthrobacter cryoconiti* strain Cr6-08 | 100 | 98.58 | NR_108846.1 |
|  |  | ANT_WA45; ANT_WA71; ANT_WB56; ANT_WB62; ANT_WB71 | *Arthrobacter stackebrandtii* strain CCM 2783 | 99 | 98.26 | NR_042258.1 |
|  |  | ANT_WB73 |  | 100 | 98.58 |  |
|  |  | ANT_WA91; ANT_WB84; ANT_WB87 | *Paeniglutamicibacter antarcticus* strain SPC26 | 100 | 98.91 | NR_115079.1 |
|  |  | ANT_WA86; ANT_WB69 | *Pseudarthrobacter oxydans* strain DSM 20119 | 100 | 99.18 | NR_026236.1 |
|  | *Cryobacterium* | ANT_WB35 | *Cryobacterium arcticum* strain SK1 | 93 | 99.49 | NR_108605.1 |
|  |  | ANT_WA20; ANT_WA21; ANT_WA24 | *Cryobacterium tepidiphilum* strain NEAU-85 | 100 | 98.02 | NR_164984.1 |
|  | *Dietzia* | ANT_WB102 | *Dietzia schimae* strain YIM 65001 | 99 | 99.54 | NR_044482.1 |
|  | *Leucobacter* | ANT_WA93 | *Leucobacter komagatae* strain IFO 15245 | 100 | 97.76 | NR_114929.1 |
|  | *Microbacterium* | ANT_WB10 | Microbacterium lacus strain A5E-52 | 100 | 98.56 | NR_041563.1 |
|  | *Rhodococcus* | ANT_WB64; ANT_WB98 | *Nocardia globerula* strain DSM 44596 | 99 | 99.26 | NR_104795.1 |
|  |  | ANT_WB48 | *Rhodococcus qingshengii* strain djl-6-2 | 99 | 98.22 | NR_115708.1 |
|  |  | ANT_WA8; ANT_WA13; ANT_WA31; ANT_WA41; ANT_WB1; ANT_WB2; ANT_WB7; ANT_WB9; ANT_WB15; ANT_WB16; ANT_WB17; ANT_WB18; ANT_WB19; ANT_WB20; ANT_WB21; ANT_WB22; ANT_WB23; ANT_WB24; ANT_WB46; ANT_WA63; ANT_WA78; ANT_WB92; ANT_WB93; ANT_WB94; ANT_WB99; ANT_WB100 | *Rhodococcus yunnanensis* strain YIM 70056 | 98 | 99.17 | NR_043009.1 |
|  |  | ANT_WB47 |  | 99 | 97.31 |  |
|  | *Salinibacterium* | ANT_WA32 | *Salinibacterium amurskyense* strain KMM 3673 | 98 | 99.86 | NR_041932.1 |
|  | *Sanguibacter* | ANT_WB5 | *Sanguibacter antarcticus* strain KOPRI 21702 | 98 | 99.93 | NR_044173.1 |
|  | *Tomitella* | ANT_WA70 | *Tomitella biformata* AHU 1821 | 99 | 98.63 | NR_112905.1 |
|  | *unclassified* | ANT_WA43 | Leifsonia rubra strain CMS 76r | 100 | 98.32 | NR_028012.1 |
|  | *unclassified Microcococaceae* | ANT_WB95; ANT_WB96; ANT_WB97 | *Neomicrococcus lactis* strain DW152 | 96 | 99.51 | NR_117033.1 |
| *Bacteroidetes* | *Chryseobacterium* | ANT_WA14; ANT_WA26; ANT_WA28; ANT_WA55; ANT_WA74; ANT_WA83; ANT_WA87; ANT_WA94; ANT_WB58 | Chryseobacterium carnis strain G81 | 97 | 97.71 | NR_126255.1 |
|  |  | ANT_WB8; ANT_WB12; ANT_WB45; ANT_WB80; ANT_WB82 | *Chryseobacterium carnis* strain G81 | 97 | 97.36 | NR_126255.1 |
|  |  | ANT_WB79 |  | 98 | 97.09 |  |
|  |  | ANT_WB57; ANT_WB78; ANT_WB81 |  | 98 | 98.20 |  |
|  |  | ANT_WA56; ANT_WA72 | *Chryseobacterium palustre* strain NBRC 104928 | 98 | 97.24 | NR_114271.1 |
|  |  | ANT_WA16; ANT_WA89; ANT_WA95 | *Chryseobacterium solincola* strain 1YB-R12 | 98 | 96.86 | NR_116343.1 |
|  | *Flavobacterium* | ANT_WA10;ANT_WA29 | *Flavobacterium degerlachei* strain NBRC 102677 | 99 | 97.93 | NR_112815.1 |
|  |  | ANT_WA11; ANT_WA12; ANT_WA15 | *Flavobacterium degerlachei* strain R-9106 | 98 | 98.96 | NR_029009.1 |
|  |  | ANT_WB4; ANT_WA30; ANT_WA73 |  | 98 | 99.31 | NR_029009.1 |
|  |  | ANT_WA9 | *Flavobacterium frigidarium* | 100 | 97.66 | NR_025020.1 |
|  |  | ANT_WA27 | *Flavobacterium piscis* strain 412R-09 | 99 | 98.41 | NR_133746.1 |
|  | *Hymenobacter* | ANT_WA1; ANT_WA2; ANT_WA3; ANT_WA7 | *Hymenobacter psychrophilus* strain BZ33r | 100 | 98.16 | NR_117214.1 |
| *Deinococcus-Thermus* | *Deinococcus* | ANT_WA4; ANT_WA5; ANT_WA6; ANT_WB39; ANT_WA54 | *Deinococcus marmoris* strain AA-63 | 100 | 98.63 | NR_042210.1 |
| *Firmicutes* | *Bacillus* | ANT_WB30 | *Bacillus frigoritolerans* | 99 | 99.93 | NR_115064.1 |
|  |  | ANT_WA51 | *Bacillus subtilis* strain DSM 10 | 100 | 100.00 | NR_027552.1 |
|  |  | ANT_WA75; ANT_WA76 | *Bacillus zhangzhouensis* strain MCCC 1A08372 | 100 | 99.79 | NR_148786.1 |
|  | *Carnobacterium* | ANT_WB67 | *Carnobacterium funditum* strain NBRC 15549 | 99 | 99.73 | NR_113773.1 |
|  | *Jeotgalibaca* | ANT_WA84; ANT_WA96; ANT_WB72 | *Jeotgalibaca dankookensis* strain EX-07 | 95 | 99.65 | NR_125553.1 |
|  |  | ANT_WA69 |  | 97 | 98.46 | NR_125553.1 |
|  |  | ANT_WB65 |  | 96 | 98.89 | NR_125553.1 |
|  | *Planococcus* | ANT_WB51; ANT_WB53 | *Planococcus halocryophilus* Or1 | 99 | 99.54 | NR_118149.2 |
|  | *Sporosarcina* | ANT_WA79 | *Filibacter limicola strain* DSM 13886 | 99 | 98.34 | NR_042024.1 |
|  | *Trichococcus* | ANT_WA18; ANT_WA19; ANT_WA67 | *Trichococcus pasteurii* strain KoTa2 | 100 | 99.60 | NR_036793.2 |
|  | *Viridibacillus* | ANT_WA17 | *Viridibacillus arenosi* strain LMG 22166 | 99 | 99.93 | NR_025628.1 |
| *Proteobacteria* | *Brevundimonas* | ANT_WA23 | *Brevundimonas denitrificans* strain TAR-002 | 99 | 99.06 | NR_133989.1 |
|  | *Lysobacter* | ANT_WA98 | *Lysobacter concretionis* strain Ko07 | 98 | 98.37 | NR_041003.1 |
|  | *Pseudomonas* | ANT_WA33;ANT_WA38; ANT_WA39 | *Pseudomonas weihenstephanensis* strain DSM 29166 | 99 | 99.87 | NR_148764.1 |
|  |  | ANT_WA97 | *Psychrobacter aquimaris* strain SW-210 | 99 | 99.25 | NR_043140.1 |
|  |  | ANT_WB31 | *Psychrobacter arcticus* strain 273-4 | 100 | 99.66 | NR_075054.1 |
|  |  | ANT_WB49 |  | 99 | 99.46 |  |
|  |  | ANT_WA40; ANT_WB27; ANT_WB28; ANT_WB29; ANT_WB32; ANT_WB42; ANT_WB50, ANT_WB70 | *Psychrobacter cryohalolentis* K5 | 100 | 99.26 | NR_075055.1 |
|  |  | ANT_WA100 | *Psychrobacter fulvigenes* strain KC 40 | 100 | 99.93 | NR_041688.1 |
|  |  | ANT_WA34; ANT_WA36; ANT_WA37; ANT_WB25; ANT_WB26; ANT_WA90 | *Psychrobacter maritimus* strain Pi2-20 | 98 | 99.59 | NR_027225.1 |
|  |  | ANT_WA35; ANT_WB41; ANT_WB54; ANT_WB68; ANT_WB74 | *Psychrobacter urativorans* strain DSM 14009 | 99 | 98.73 | NR_042221.1 |
|  | *Pusillimonas* | ANT_WB101 | *Pusillimonas ginsengisoli* strain DCY25 | 95 | 98.99 | NR_116103.1 |
|  | *Stenotrophomonas* | ANT_WB33; ANT_WB34 | *Stenotrophomonas humi* strain R-32729 | 100 | 97.92 | NR_042568.1 |
