# Supplementary material for: Insight Into Ecology, Metabolic Potential, and the Taxonomic Composition of Bacterial Communities in the Periodic Water Pond on King George Island (Antarctica)
Source: Front Microbiol. 2021 Oct 8;12:708607. doi: 10.3389/fmicb.2021.708607 (PMC8531505; doi:10.3389/fmicb.2021.708607)
Supplement: Supplementary Table 4 — Summary information on the amplicons used for the pond comparison. [file Table_4.docx]

Table S4 Summary information on the amplicons used for the pond comparison.

| **No** | **NCBI SRR** | **Geographic coordinate** | **Sample** | **Distance from the Pond [km]** | **Isolation Source** | **Cluster (based on the Bray-Curtis dissimilarity matrix)** | **Cluster according to isolation source** |
| --- | --- | --- | --- | --- | --- | --- | --- |
| 1 | - | 62.234655 S 58.472860 W | BT1 | 0 | Pond | 13 | P13 |
| 2 | - | 62.234655 S 58.472860 W | BT2 | 0 | Pond | 13 | P13 |
| 3 | SRR8065446 | 76.91 S 160.93 E | SR1 | 4304.119855 | Sandstone rock | 6 | SR6 |
| 4 | SRR8065489 | 75.70 S 162.06 E | SR2 | 4408.52304 | Sandstone rock | 2 | SR2 |
| 5 | SRR8065491 | 75.94 S 159.71 E | SR3 | 4414.486504 | Sandstone rock | 2 | SR2 |
| 6 | SRR8065467 | 75.94 S 159.64 E | SR4 | 4415.338265 | Sandstone rock | 6 | SR6 |
| 7 | SRR8065484 | 75.75 S 161.06 E | SR5 | 4416.480021 | Sandstone rock | 2 | SR2 |
| 8 | SRR8065477 | 75.80 S 159.18 E | SR6 | 4434.844913 | Sandstone rock | 2 | SR2 |
| 9 | SRR8065464 | 75.51 S 159.81 E | SR7 | 4455.963369 | Sandstone rock | 2 | SR2 |
| 10 | SRR8065486 | 75.50 S 159.67 E | SR8 | 4458.701465 | Sandstone rock | 6 | SR6 |
| 11 | SRR8065443 | 74.55 S 162.06 E | SR9 | 4521.42557 | Sandstone rock | 13 | SR13 |
| 12 | SRR8065479 | 74.17 S 162.51 E | SR10 | 4552.545376 | Sandstone rock | 6 | SR6 |
| 13 | SRR8065462 | 74.16 S 162.42 E | SR11 | 4554.801134 | Sandstone rock | 4 | SR4 |
| 14 | SRR8065450 | 74.08 S 162.86 E | SR12 | 4556.411815 | Sandstone rock | 6 | SR6 |
| 15 | SRR8065468 | 74.04 S 162.61 E | SR13 | 4563.913739 | Sandstone rock | 6 | SR6 |
| 16 | SRR8065452 | 74.03 S 162.62 E | SR14 | 4564.754854 | Sandstone rock | 6 | SR6 |
| 17 | SRR8065456 | 73.49 S 163.91 E | SR15 | 4598.845042 | Sandstone rock | 2 | SR2 |
| 18 | SRR8916032 | 62.183417 S 58.442667 W | S1 | 5.908536247 | Antarctic soil | 2 | S2 |
| 19 | SRR11004816 | 62.23 S 58.78 W | S2 | 15.91960379 | Antarctic soil | 6 | S6 |
| 20 | SRR11004820 | 62.23 S 58.78 W | S3 | 15.91960379 | Antarctic soil | 2 | S2 |
| 21 | SRR6677372 | 62.18 S 58.84 W | S4 | 19.98181404 | Antarctic soil | 1 | S1 |
| 22 | SRR6679562 | 62.18 S 58.85 W | S5 | 20.47632992 | Antarctic soil | 2 | S2 |
| 23 | SRR6679589 | 62.18 S 58.86 W | S6 | 20.97200279 | Antarctic soil | 4 | S4 |
| 24 | SRR6679586 | 62.18 S 58.87 W | S7 | 21.4687525 | Antarctic soil | 9 | S9 |
| 25 | SRR6679584 | 62.18 S 58.88 W | S8 | 21.96650598 | Antarctic soil | 11 | S11 |
| 26 | SRR6679583 | 62.18 S 58.89 W | S9 | 22.4651965 | Antarctic soil | 9 | S9 |
| 27 | SRR6677377 | 62.19 S 58.91 W | S10 | 23.19836799 | Antarctic soil | 7 | S7 |
| 28 | SRR6677378 | 62.19 S 58.92 W | S11 | 23.70499806 | Antarctic soil | 10 | S10 |
| 29 | SRR6677368 | 62.17 S 58.91 W | S12 | 23.78098122 | Antarctic soil | 1 | S1 |
| 30 | SRR6679579 | 62.21 S 58.93 W | S13 | 23.84776421 | Antarctic soil | 7 | S7 |
| 31 | SRR6679567 | 62.20 S 58.93 W | S14 | 24.00489561 | Antarctic soil | 6 | S6 |
| 32 | SRR6677375 | 62.19 S 58.93 W | S15 | 24.21212561 | Antarctic soil | 10 | S10 |
| 33 | SRR6679582 | 62.21 S 58.94 W | S16 | 24.36261373 | Antarctic soil | 3 | S3 |
| 34 | SRR6677357 | 62.20 S 58.94 W | S17 | 24.51661322 | Antarctic soil | 5 | S5 |
| 35 | SRR6677366 | 62.17 S 58.93 W | S18 | 24.77154872 | Antarctic soil | 10 | S10 |
| 36 | SRR6679581 | 62.21 S 58.95 W | S19 | 24.87760276 | Antarctic soil | 4 | S4 |
| 37 | SRR6677354 | 62.20 S 58.95 W | S20 | 25.0286015 | Antarctic soil | 1 | S1 |
| 38 | SRR6677376 | 62.15 S 58.92 W | S21 | 25.03180777 | Antarctic soil | 1 | S1 |
| 39 | SRR6677365 | 62.22 S 58.96 W | S22 | 25.29266448 | Antarctic soil | 1 | S1 |
| 40 | SRR6677373 | 62.15 S 58.93 W | S23 | 25.51320554 | Antarctic soil | 7 | S7 |
| 41 | SRR6679590 | 62.24 S 58.97 W | S24 | 25.75656271 | Antarctic soil | 4 | S4 |
| 42 | SRR6677364 | 62.22 S 58.97 W | S25 | 25.80973562 | Antarctic soil | 10 | S10 |
| 43 | SRR6677361 | 62.18 S 58.96 W | S26 | 25.97772103 | Antarctic soil | 7 | S7 |
| 44 | SRR6677374 | 62.15 S 58.94 W | S27 | 25.99603935 | Antarctic soil | 13 | S13 |
| 45 | SRR6679585 | 62.20 S 58.97 W | S28 | 26.05332619 | Antarctic soil | 13 | S13 |
| 46 | SRR6677360 | 62.17 S 58.96 W | S29 | 26.26413731 | Antarctic soil | 1 | S1 |
| 47 | SRR6677379 | 62.24 S 58.98 W | S30 | 26.27438169 | Antarctic soil | 1 | S1 |
| 48 | SRR6677363 | 62.22 S 58.98 W | S31 | 26.32684808 | Antarctic soil | 1 | S1 |
| 49 | SRR6679565 | 62.18 S 58.97 W | S32 | 26.48208038 | Antarctic soil | 4 | S4 |
| 50 | SRR6677369 | 62.20 S 58.98 W | S33 | 26.56603372 | Antarctic soil | 1 | S1 |
| 51 | SRR6679560 | 62.17 S 58.97 W | S34 | 26.76326312 | Antarctic soil | 4 | S4 |
| 52 | SRR6679588 | 62.24 S 58.99 W | S35 | 26.79220575 | Antarctic soil | 4 | S4 |
| 53 | SRR6679564 | 62.18 S 58.98 W | S36 | 26.98697436 | Antarctic soil | 9 | S9 |
| 54 | SRR6677370 | 62.20 S 58.99 W | S37 | 27.07895392 | Antarctic soil | 5 | S5 |
| 55 | SRR6677362 | 62.17 S 58.98 W | S38 | 27.26311404 | Antarctic soil | 10 | S10 |
| 56 | SRR6677349 | 62.21 S 59.01 W | S39 | 27.97003412 | Antarctic soil | 5 | S5 |
| 57 | SRR6679577 | 62.21 S 59.02 W | S40 | 28.4857952 | Antarctic soil | 4 | S4 |
| 58 | SRR6679580 | 62.21 S 59.03 W | S41 | 29.00164345 | Antarctic soil | 3 | S3 |
| 59 | SRR8915552 | 62.673303 S 61.107672 W | S42 | 143.9903546 | Antarctic soil | 4 | S4 |
| 60 | SRR8915978 | 61.220886 S 55.139694 W | S43 | 208.5916375 | Antarctic soil | 1 | S1 |
| 61 | SRR8915964 | 64.810347 S 63.778572 W | S44 | 388.6167683 | Antarctic soil | 7 | S7 |
| 62 | SRR8915692 | 65.32815 S 64.160033 W | S45 | 442.8033476 | Antarctic soil | 7 | S7 |
| 63 | SRR8915966 | 60.706633 S 45.592483 W | S46 | 703.5348115 | Antarctic soil | 7 | S7 |
| 64 | SRR8916009 | 60.706633 S 45.592483 W | S47 | 703.5348115 | Antarctic soil | 4 | S4 |
| 65 | SRR8916021 | 67.606533 S 68.207367 W | S48 | 751.417984 | Antarctic soil | 7 | S7 |
| 66 | SRR8915548 | 67.601197 S 68.350233 W | S49 | 755.0502182 | Antarctic soil | 2 | S2 |
| 67 | SRR8915545 | 67.72455 S 68.40285 W | S50 | 766.6968702 | Antarctic soil | 2 | S2 |
| 68 | SRR8916019 | 71.844417 S 68.225783 W | S51 | 1145.855976 | Antarctic soil | 13 | S13 |
| 69 | SRR7881185 | 71.9 S 68.2 W | S52 | 1151.015214 | Antarctic soil | 4 | S4 |
| 70 | SRR7881193 | 71.9 S 68.2 W | S53 | 1151.015214 | Antarctic soil | 13 | S13 |
| 71 | SRR8915576 | 74.83025 S 71.550683 W | S54 | 1490.12658 | Antarctic soil | 10 | S10 |
| 72 | SRR8915860 | 74.844317 S 71.6016 W | S55 | 1492.193528 | Antarctic soil | 5 | S5 |
| 73 | SRR8173711 | 72.64610 S 3.76293 W | S56 | 2485.537587 | Antarctic soil | 4 | S4 |
| 74 | SRR8173666 | 72.01518 S 3.38577 W | S57 | 2500.710629 | Antarctic soil | 4 | S4 |
| 75 | SRR8173651 | 72.13352 S 3.27808 W | S58 | 2503.738102 | Antarctic soil | 9 | S9 |
| 76 | SRR8173729 | 71.89484 S 3.23095 W | S59 | 2506.77183 | Antarctic soil | 13 | S13 |
| 77 | SRR8173710 | 71.89420 S 3.22727 W | S60 | 2506.902962 | Antarctic soil | 13 | S13 |
| 78 | SRR8173655 | 71.48090 S 3.20211 W | S61 | 2510.892606 | Antarctic soil | 13 | S13 |
| 79 | SRR8173730 | 71.48071 S 3.20102 W | S62 | 2510.932607 | Antarctic soil | 2 | S2 |
| 80 | SRR8173715 | 71.90226 S 2.97322 W | S63 | 2515.609503 | Antarctic soil | 9 | S9 |
| 81 | SRR8173663 | 72.04877 S 2.79029 W | S64 | 2520.89892 | Antarctic soil | 9 | S9 |
| 82 | SRR8173662 | 71.76181 S 2.82512 W | S65 | 2521.773645 | Antarctic soil | 9 | S9 |
| 83 | SRR8173692 | 71.75825 S 2.81686 W | S66 | 2522.088454 | Antarctic soil | 3 | S3 |
| 84 | SRR8173658 | 71.75825 S 2.80942 W | S67 | 2522.346764 | Antarctic soil | 9 | S9 |
| 85 | SRR8173695 | 72.05402 S 2.72549 W | S68 | 2523.080536 | Antarctic soil | 13 | S13 |
| 86 | SRR8173697 | 72.03431 S 2.54063 E | S69 | 2702.845582 | Antarctic soil | 4 | S4 |
| 87 | SRR8173623 | 72.00798 S 2.54828 E | S70 | 2703.520981 | Antarctic soil | 7 | S7 |
| 88 | SRR8173613 | 72.00192 S 2.57425 E | S71 | 2704.500172 | Antarctic soil | 3 | S3 |
| 89 | SRR8173721 | 72.01083 S 2.60013 E | S72 | 2705.238205 | Antarctic soil | 2 | S2 |
| 90 | SRR8173627 | 72.02589 S 2.63330 E | S73 | 2706.125514 | Antarctic soil | 11 | S11 |
| 91 | SRR8173684 | 72.02492 S 2.63511 E | S74 | 2706.202416 | Antarctic soil | 7 | S7 |
| 92 | SRR8173644 | 72.03275 S 2.67286 E | S75 | 2707.359473 | Antarctic soil | 7 | S7 |
| 93 | SRR8173615 | 72.03342 S 2.67658 E | S76 | 2707.475069 | Antarctic soil | 13 | S13 |
| 94 | SRR8173643 | 72.03738 S 2.75836 E | S77 | 2710.187204 | Antarctic soil | 7 | S7 |
| 95 | SRR3051872 | 84.240 S 153.694 W | S78 | 3204.613135 | Antarctic soil | 8 | S8 |
| 96 | SRR8654257 | 77.7304 S 162.323 E | S79 | 4207.851265 | Antarctic soil | 12 | S12 |
| 97 | SRR1030781 | 77.6197 S 163.218683 E | S80 | 4207.864494 | Antarctic soil | 5 | S5 |
| 98 | SRR8654469 | 77.6397 S 162.9401 E | S81 | 4209.285209 | Antarctic soil | 12 | S12 |
| 99 | SRR8654432 | 77.6337 S 162.9311 E | S82 | 4209.969775 | Antarctic soil | 12 | S12 |
| 100 | SRR8654260 | 77.6336 S 162.8644 E | S83 | 4210.776155 | Antarctic soil | 12 | S12 |
| 101 | SRR8754893 | 77.87202 S 160.4954 E | S84 | 4214.986313 | Antarctic soil | 6 | S6 |
| 102 | SRR10136532 | 62.16352 S 58.46270 W | F1 | 7.927392619 | Feces | 3 | F3 |
| 103 | SRR10136533 | 62.16352 S 58.46270 W | F2 | 7.927392619 | Feces | 3 | F3 |
| 104 | SRR10136534 | 62.16352 S 58.46270 W | F3 | 7.927392619 | Feces | 3 | F3 |
| 105 | SRR7614687 | 68.659 S 77.869 E | F4 | 5053.762883 | Feces | 11 | F11 |
| 106 | SRR7614693 | 68.578 S 77.870 E | F5 | 5061.819937 | Feces | 11 | F11 |
| 107 | SRR7614682 | 68.498 S 78.073 E | F6 | 5073.478515 | Feces | 11 | F11 |
| 108 | SRR7614688 | 68.458 S 78.170 E | F7 | 5079.22707 | Feces | 11 | F11 |
| 109 | SRR7614689 | 68.431 S 78.396 E | F8 | 5086.031077 | Feces | 8 | F8 |
| 110 | SRR7614692 | 68.431 S 78.396 E | F9 | 5086.031077 | Feces | 11 | F11 |
| 111 | SRR1254130 | 84.240 S 153.694 W | L1 | 3204.613135 | Lake | 13 | L13 |
| 112 | SRR3051882 | 84.240 S 153.694 W | L2 | 3204.613135 | Lake | 13 | L13 |
| 113 | SRR8799241 | 71.96589 S 23.33311 E | L3 | 3387.461389 | Lake | 12 | L12 |
| 114 | SRR10432411 | 77.733333 S 162.1666 W | L4 | 3639.388008 | Lake | 11 | L11 |
| 115 | SRR8790579 | 77.716666 S 162.2833 E | L5 | 4209.642462 | Lake | 3 | L3 |
| 116 | SRR3997475 | 62.210800 S 58.365083 W | MS1 | 6.182977484 | Marine sediment | 13 | MS13 |
| 117 | SRR3997474 | 62.173883 S 58.394716 W | MS2 | 7.879220829 | Marine sediment | 4 | MS4 |
| 118 | SRR3997473 | 62.158600 S 58.423750 W | MS3 | 8.832196456 | Marine sediment | 10 | MS10 |
| 119 | SRR3997476 | 62.272583 S 58.300833 W | MS4 | 9.853615429 | Marine sediment | 9 | MS9 |
| 120 | SRR3997471 | 62.141333 S 58.475883 W | MS5 | 10.37809927 | Marine sediment | 5 | MS5 |
| 121 | SRR3997467 | 62.292600 S 58.303500 W | MS6 | 10.87800253 | Marine sediment | 9 | MS9 |
| 122 | SRR3997472 | 62.138250 S 58.402250 W | MS7 | 11.32847131 | Marine sediment | 5 | MS5 |
| 123 | SRR3997465 | 62.266633 S 58.224950 W | MS8 | 13.31846992 | Marine sediment | 10 | MS10 |
| 124 | SRR3997468 | 62.287616 S 58.240583 W | MS9 | 13.38643061 | Marine sediment | 10 | MS10 |
| 125 | SRR3997463 | 62.103883 S 58.410550 W | MS10 | 14.8966002 | Marine sediment | 10 | MS10 |
| 126 | SRR3997470 | 62.104383 S 58.397133 W | MS11 | 15.00951666 | Marine sediment | 10 | MS10 |
| 127 | SRR3997469 | 62.098883 S 58.437500 W | MS12 | 15.20834869 | Marine sediment | 5 | MS5 |
| 128 | SRR3997464 | 62.274700 S 58.169666 W | MS13 | 16.31451251 | Marine sediment | 4 | MS4 |
| 129 | SRR3997462 | 62.08 S 58.37 W | MS14 | 18.00743157 | Marine sediment | 5 | MS5 |
| 130 | SRR2827648 | 62.53536 S 59.08836 W | MS15 | 46.09134028 | Marine sediment | 12 | MS12 |
| 131 | SRR6704963 | 62.64 S 58.97 W | MS16 | 51.8243255 | Marine sediment | 12 | MS12 |
| 132 | SRR6704962 | 62.19 S 57.30 W | MS17 | 61.00115579 | Marine sediment | 12 | MS12 |
| 133 | SRR6704966 | 62.19 S 57.27 W | MS18 | 62.55121967 | Marine sediment | 7 | MS7 |
| 134 | SRR6704965 | 62.10 S 56.61 W | MS19 | 97.86084759 | Marine sediment | 6 | MS6 |
| 135 | SRR6704964 | 62.87 S 59.92 W | MS20 | 102.4284028 | Marine sediment | 6 | MS6 |
| 136 | SRR5535667 | 62.06 S 60.71 W | MS21 | 117.8253525 | Marine sediment | 6 | MS6 |
| 137 | SRR5535670 | 63.18 S 60.67 W | MS22 | 153.6057464 | Marine sediment | 2 | MS2 |
| 138 | SRR5595784 | 77.851 S 160.664 E | MS23 | 4215.156445 | Marine sediment | 13 | MS13 |
| 139 | SRR5595767 | 77.800 S 160.671 E | MS24 | 4220.057891 | Marine sediment | 8 | MS8 |
| 140 | SRR7614678 | 76.792 S 164.733 E | MS25 | 4268.069813 | Marine sediment | 13 | MS13 |
| 141 | SRR7614679 | 68.577 S 77.965 E | MS26 | 5063.656451 | Marine sediment | 13 | MS13 |
| 142 | SRR7614672 | 68.523 S 78.064 E | MS27 | 5070.829347 | Marine sediment | 13 | MS13 |
| 143 | SRR8185224 | 71.49594 S 3.235472 W | MP1 | 2509.591118 | Meltwater pond | 13 | MP13 |
| 144 | SRR8185254 | 71.49594 S 3.235472 W | MP2 | 2509.591118 | Meltwater pond | 7 | MP7 |
| 145 | SRR8185244 | 71.99647 S 2.623056 E | MP3 | 2706.246897 | Meltwater pond | 12 | MP12 |
| 146 | SRR8185270 | 71.99647 S 2.623056 E | MP4 | 2706.246897 | Meltwater pond | 6 | MP6 |
| 147 | SRR8185215 | 71.99661 S 2.623139 E | MP5 | 2706.247478 | Meltwater pond | 7 | MP7 |
| 148 | SRR8185268 | 71.99661 S 2.623139 E | MP6 | 2706.247478 | Meltwater pond | 13 | MP13 |
| 149 | SRR8185183 | 72.00894 S 2.636389 E | MP7 | 2706.500713 | Meltwater pond | 13 | MP13 |
| 150 | SRR8185235 | 72.03164 S 2.657333 E | MP8 | 2706.850049 | Meltwater pond | 13 | MP13 |
| 151 | SRR8185246 | 72.03164 S 2.657333 E | MP9 | 2706.850049 | Meltwater pond | 12 | MP12 |
| 152 | SRR8185237 | 72.02947 S 2.659694 E | MP10 | 2706.964771 | Meltwater pond | 6 | MP6 |
| 153 | SRR8185240 | 72.03008 S 2.660947 E | MP11 | 2706.997593 | Meltwater pond | 8 | MP8 |
| 154 | SRR8185200 | 72.02617 S 2.796528 E | MP12 | 2711.663312 | Meltwater pond | 8 | MP8 |
| 155 | SRR8185272 | 72.02617 S 2.796528 E | MP13 | 2711.663312 | Meltwater pond | 12 | MP12 |
| 156 | SRR8185186 | 71.03531 S 2.542944 E | MP14 | 2720.718882 | Meltwater pond | 12 | MP12 |
| 157 | SRR8185228 | 71.03531 S 2.542944 E | MP15 | 2720.718882 | Meltwater pond | 12 | MP12 |
| 158 | SRR8185191 | 71.0355 S 2.54425 E | MP16 | 2720.761537 | Meltwater pond | 13 | MP13 |
| 159 | SRR8185226 | 71.0355 S 2.54425 E | MP17 | 2720.761537 | Meltwater pond | 2 | MP2 |
| 160 | SRR8185190 | 71.00822 S 2.632583 E | MP18 | 2724.449635 | Meltwater pond | 13 | MP13 |
| 161 | SRR8185242 | 71.00894 S 2.636389 E | MP19 | 2724.570577 | Meltwater pond | 12 | MP12 |
| 162 | SRR8185236 | 71.02406 S 2.651167 E | MP20 | 2724.792016 | Meltwater pond | 8 | MP8 |
| 163 | SRR11940279 | 62.628633 S 60.356667 W | SW1 | 106.3777644 | Seawater | 2 | SW2 |
| 164 | SRR11940278 | 62.659267 S 60.368867 W | SW2 | 108.344663 | Seawater | 4 | SW4 |
| 165 | SRR11940297 | 62.659267 S 60.368867 W | SW3 | 108.344663 | Seawater | 7 | SW7 |
| 166 | SRR12301953 | 62.659267 S 60.368867 W | SW4 | 108.344663 | Seawater | 4 | SW4 |
| 167 | SRR11940287 | 62.6391 S 60.398533 W | SW5 | 108.8043692 | Seawater | 4 | SW4 |
| 168 | SRR11940302 | 62.6391 S 60.398533 W | SW6 | 108.8043692 | Seawater | 7 | SW7 |
| 169 | SRR12301951 | 62.6391 S 60.398533 W | SW7 | 108.8043692 | Seawater | 13 | SW13 |
| 170 | SRR12301955 | 62.657083 S 60.38795 W | SW8 | 109.1273388 | Seawater | 4 | SW4 |
| 171 | SRR12301956 | 62.657083 S 60.38795 W | SW9 | 109.1273388 | Seawater | 6 | SW6 |
| 172 | SRR11940283 | 62.65815 S 60.440517 W | SW10 | 111.6220242 | Seawater | 4 | SW4 |
| 173 | SRR11940276 | 62.646533 S 60.9735 W | SW11 | 136.5472902 | Seawater | 4 | SW4 |
| 174 | SRR10953107 | 64.42125 S 63.2829 W | SW12 | 341.5269731 | Seawater | 2 | SW2 |
| 175 | SRR10953108 | 64.6438 S 62.88905 W | SW13 | 346.2141818 | Seawater | 6 | SW6 |
| 176 | SRR10541915 | 64.850218 S 63.582774 W | SW14 | 385.3471465 | Seawater | 1 | SW1 |
| 177 | SRR10541917 | 64.850218 S 63.582774 W | SW15 | 385.3471465 | Seawater | 1 | SW1 |
| 178 | SRR10541922 | 64.850218 S 63.582774 W | SW16 | 385.3471465 | Seawater | 1 | SW1 |
| 179 | SRR10541923 | 64.850218 S 63.582774 W | SW17 | 385.3471465 | Seawater | 3 | SW3 |
| 180 | SRR10953106 | 64.7794833 S 64.07255 W | SW18 | 396.2435422 | Seawater | 6 | SW6 |
| 181 | SRR10953112 | 64.7794833 S 64.07255 W | SW19 | 396.2435422 | Seawater | 6 | SW6 |
| 182 | SRR10953117 | 64.7794833 S 64.07255 W | SW20 | 396.2435422 | Seawater | 4 | SW4 |
| 183 | SRR10953113 | 64.7834833 S 64.7776 W | SW21 | 421.6909202 | Seawater | 2 | SW2 |
| 184 | SRR10953115 | 64.7834833 S 64.7776 W | SW22 | 421.6909202 | Seawater | 9 | SW9 |
| 185 | SRR10953116 | 64.7834833 S 64.7776 W | SW23 | 421.6909202 | Seawater | 11 | SW11 |
| 186 | SRR10953110 | 64.9259333 S 65.3827 W | SW24 | 453.9076793 | Seawater | 3 | SW3 |
| 187 | SRR3310327 | 67.34 S 68.13 W | SW25 | 727.3898176 | Seawater | 11 | SW11 |
| 188 | SRR3310330 | 67.34 S 68.13 W | SW26 | 727.3898176 | Seawater | 8 | SW8 |
| 189 | SRR3310332 | 67.34 S 68.13 W | SW27 | 727.3898176 | Seawater | 6 | SW6 |
| 190 | SRR11196300 | 75.12672 S 164.5333 W | SW28 | 3858.563925 | Seawater | 2 | SW2 |
| 191 | SRR11196306 | 74.75716 S 166.8105 W | SW29 | 3935.508874 | Seawater | 8 | SW8 |
| 192 | SRR11196324 | 74.18770 S 170.9092 W | SW30 | 4068.511959 | Seawater | 1 | SW1 |
| 193 | SRR11196288 | 74.00000 S 175.0890 W | SW31 | 4172.910804 | Seawater | 3 | SW3 |
| 194 | SRR10012272 | 68.35 S 77.58 E | SW32 | 5079.101347 | Seawater | 6 | SW6 |
