# Supplementary material for: Insight Into Ecology, Metabolic Potential, and the Taxonomic Composition of Bacterial Communities in the Periodic Water Pond on King George Island (Antarctica)
Source: Front Microbiol. 2021 Oct 8;12:708607. doi: 10.3389/fmicb.2021.708607 (PMC8531505; doi:10.3389/fmicb.2021.708607)
Supplement: Supplementary Table 5 — Alpha-diversity of the samples compared in this study. [file Table_5.docx]

Table S5 Alpha-diversity of the samples compared in this study.

| **Isolation source** | **Observed** | **Chao1** | **Shannon** | **(inverted) Simpson** |
| --- | --- | --- | --- | --- |
| Pond | 964.50±106.77 | 1241.85±80.85 | 4.46±0.37 | 0.97±0.01 |
| Antarctic soil | 399.62±279.46 | 599.00±387.16 | 3.16±0.96 | 0.87±0.14 |
| Feces | 505.56±204.35 | 696.01±281.59 | 2.9±0.34 | 0.88±0.03 |
| Lake | 720.00±300.75 | 959.01±278.73 | 2.96±0.77 | 0.82±0.14 |
| Marine sediment | 335.30±315.04 | 460.99±415.48 | 2.15±1.78 | 0.52±0.39 |
| Meltwater pond | 449.35±111.11 | 672.48±157.34 | 2.84±0.98 | 0.72±0.21 |
| Sandstone rock | 216.67±106.27 | 364.38±171.49 | 2.54±0.70 | 0.8±0.11 |
| Seawater | 555.69±152.90 | 760.68±165.85 | 3.19±0.92 | 0.82±0.16 |
