# Supplementary material for: Insight Into Ecology, Metabolic Potential, and the Taxonomic Composition of Bacterial Communities in the Periodic Water Pond on King George Island (Antarctica)
Source: Front Microbiol. 2021 Oct 8;12:708607. doi: 10.3389/fmicb.2021.708607 (PMC8531505; doi:10.3389/fmicb.2021.708607)
Supplement: Supplementary File 1 — Extended description of the used methods. [file Data_Sheet_1.docx]

**Supplementary File**

**Genomic DNA extraction, sequencing and analysis of 16S rRNA gene sequences**

Amplification reaction of 16S rRNA gene was carried out in 25 μl of the reagent mixture to PCR using 100 pmols of each primer (27F: 5’-AGAGTTTGATCCTGGCTCAG-3’ and 1492R: 5’-GGTTACCTTGTTACGACTT-3), 0.5-1 μl of DNA, 0.5 μl of the dNTP mixture with a final concentration of 10 mM (each), buffer and Mg^2+^ ion solution (supplied by the polymerase manufacturer) and 1-2.5 μl of Taq polymerase. The reaction was performed with the use of Mastercycler nexus GX2 thermocycler from the Eppendorf company. After 5-minute denaturation of DNA at 95°C, 35 cycles were performed, including 20 cycles consisting of the following stages: denaturation (95°C, 50 s), primer annealing (53°C, 50 s), DNA synthesis (72°C, 1.5 min), 15 cycles consisting of the following stages: denaturation (95°C, 30 s), primer annealing (46°C, 30 s), DNA synthesis (72°C, 1.5 min). The last reaction cycle ended with a 10-minute synthesis (72°C) to fill the single-stranded ends of the amplified fragments. The PCR mix was then purified by adding ExoI (1.5 μl) and FastAP (3 μl) enzymes to the reaction mixture (15 μl) and then mixed and incubated at 37°C for 30 minutes. After the incubation time, the enzymes were deactivated using 85°C and 30 minutes incubation.

**Metagenomic DNA isolation from water samples and sequencing**

The amplification reactions were carried out in 25 μl of the reagent mixture to PCR using 10 pmols of each primer, 0.5-1 μl of DNA matrix, 0.5 μl of the dNTP mixture with a final concentration of 10 mM (each), buffer and Mg2+ ion solution (supplied by the polymerase manufacturer) and 1-2.5 μl of KAPA polymerase. The reaction was carried out with the use of Mastercycler nexus GX2 thermocycler from Eppendorf. After 5-minute denaturation of DNA at 95°C, 35 cycles were performed, consisting of the following stages: denaturation (95°C, 30 s), primer annealing (53°C - V3-4 primers), DNA synthesis (72°C, 1.5 min). The last reaction cycle ended with a 10-minute synthesis (72°C) to fill the single-stranded ends of the amplified fragments. Then samples were sequenced in a paired-end mode using a v3 chemistry kit with the Illumina MiSeq instrument (Illumina, San Diego, CA, USA) by the DNA Sequencing and Oligonucleotide Synthesis Laboratory—oligo.pl (Institute of Biochemistry and Biophysics, Polish Academy of Sciences).
